# Effects of a 4-week combined breathing control and self-talk intervention on neural activity and coping skills in athletes: a randomized control trial

**DOI:** 10.1186/s40359-026-04814-w

**Published:** 2026-06-05

**Authors:** Kittichai Tharawadeepimuk, Ekarat Onnom, Ampika Nanbancha

**Affiliations:** College of Sports Science and Technology, Mahidol University, Nakhonpathom, Thailand

**Keywords:** Breathing control, Self-talk, Electroencephalogram (EEG), Athletic Coping Skills Inventory-28 (ACSI-28), Cognitive control

## Abstract

**Background:**

Effective coping skills are essential for athletes aiming to achieve peak performance. Breathing control and self-talk are recognized as commonly used psychological interventions to support coping. However, their combined effects on neurophysiological and psychological markers have not been examined.

**Objective:**

To evaluate the effects of a 4-week combined breathing control and self-talk intervention on electroencephalogram (EEG) activity and coping skills in elite national-level athletes.

**Method:**

Twenty-six athletes (11 swimmers and 15 gymnasts) were randomized to an experimental or control group. The intervention consisted of 16 sessions over four weeks (four days per week). Resting-state EEG and Athletic Coping Skills Inventory-28 (ACSI-28) scores were collected pre- and post-intervention. EEG power spectral density (PSD) across frequency bands was analyzed using generalized linear mixed models, and ACSI-28 subscales were assessed using repeated measures ANOVA.

**Result:**

Significant group differences were found in theta (*p* < .001) and beta (*p* = .048) power. The experimental group showed increased theta power in the prefrontal area (*p* < .001) and decreased beta power in the frontal area (*p* = .003). Improvements were also observed in ACSI-28 subscales, including coping with adversity (*p* = .018), concentration (*p* = .027), confidence and achievement motivation (*p* = .039), goal setting/mental preparation (*p* = .009), and peaking under pressure (*p* = .043).

**Conclusion:**

This combined intervention of breathing control and self-talk was associated with changes in neurophysiological markers and psychological coping skills. EEG findings suggest potential changes in neural processes potentially related to anxiety regulation and attention. However, these should be interpreted as indirect neural correlates rather than direct markers of psychological states. Overall, these findings provide preliminary evidence that the combined intervention may represent a promising approach for supporting pre-competition psychological readiness in athletes.

**Trial registration:**

ClinicalTrials.gov NCT05042518 (09/01/2021). Prospectively registered.

**Supplementary Information:**

The online version contains supplementary material available at 10.1186/s40359-026-04814-w.

## Introduction

The most critical and meaningful aspect for athletes in competition is achieving peak performance during the event. Optimal performance is influenced by several key factors, including physical readiness (e.g., overcoming physical fatigue), competitive experience, and cognitive processes such as decision-making and anxiety regulation, as well as coping with psychological pressure, unpleasant emotions, and mental fatigue [1, 2]. Athletes are frequently confronted with numerous stressful situations throughout their training and competitive endeavors. During competitions, they may experience adverse feelings, including fear of failure, self-doubt, and anxiety related to interactions with their coach, while training sessions can induce exhaustion, fatigue, and concerns about injury. Therefore, the capability to cope with these stressors is a crucial factor in preventing diminished performance and potential failure [3]. Lazarus and Folkman (1984) defined coping as “the cognitive and behavioral efforts” used to manage specific external and internal demands [4]. Given the importance of psychological coping in athletic performance, cognitive processes are often an overlooked component of athletic preparation. Insufficient emphasis on cognitive readiness can hinder an athlete’s ability to manage competitive situations and achieve peak performance [5–7].

Optimizing cognitive processes and psychological factors requires systematic psychological training [8]. Psychological skill or mental training can influence athletes’ performances, particularly their attention and focus [9]. Furthermore, various mental training techniques and strategies consistently demonstrate a positive influence on performance and mental skills [10–13]. One example of these is self-talk, a multidimensional psychological strategy. Self-talk refers to the use of positive or negative open or closed statements that individuals direct toward themselves. These statements serve both instructional and motivational purposes [14]. The efficacy of this strategy is well-documented, with research demonstrating its ability to improve motor performance [12], self-efficacy [15], self-confidence [16], and motivation [14]. Additionally, self-talk has been found to reduce anxiety [17] and help manage attention and focus [18]. Consequently, when faced with challenges in competition, athletes use self-talk as a self-regulation strategy to cope with the mental demands of the sport [19]. This strategy can be used either independently or in combination with other psychological skill training [20].

Breathing control training has also been recognized as a fundamental psychological intervention in pre-competition and warm-up routines, playing a crucial role in optimizing athletic performance. This method helps athletes cope with stress, reduce anxiety, maintain cognitive clarity, and sustain attentional focus under competitive pressure [10, 21]. Breathing techniques modulate autonomic nervous system activity, thereby facilitating physiological and psychological readiness. This physiological readiness, in turn, can improve motor performance, reaction time, and decision-making [22–24]. Integrating breathing techniques with therapeutic interventions significantly enhances heart rate variability, suggesting that breathing exercises are valuable tools for improving physiological regulation in individuals with high anxiety sensitivity [25]. Beyond its psychological effects, breathing has also been shown to modulate neural activity. For instance, slow-symmetric breathing with breath-hold phases leads to meaningful alterations in electroencephalography (EEG) parameters, such as beta band power [26]. This finding is consistent with several studies suggesting that EEG provides a more comprehensive and objective evaluation of psychological interventions by offering neural correlates, thereby allowing for an understanding of their impact [27–30].

Although breathing control and self-talk have typically been studied independently, they target complementary mechanisms of self-regulation. Self-talk primarily influences cognitive and emotional domains, including attentional focus [18], motivation [14], self-efficacy [15], self-confidence [16], and anxiety regulation [17]. In contrast, breathing control directly modulates physiological arousal, supporting emotional stability and cognitive clarity through autonomic regulation [10, 21–24]. Controlled breathing also enhances physiological readiness [25], such as heart rate variability, and influences neural activity [26] measured by EEG. Integrating these techniques may therefore provide a more comprehensive self-regulation strategy with self-talk facilitating top-down cognitive control and breathing control providing bottom-up physiological regulation. This combination may have a potentiate effect, whereby increased physiological calmness enhances the effectiveness of cognitive strategies, and improved cognitive framing strengthens the benefits of breathing control. Despite this conceptual rationale, empirical evidence examining this combined technique with EEG and psychological measures in competitive setting remains limited.

Crucially, while both techniques independently demonstrate psychological and physiological benefits, research examining their combined application using EEG and the Athletic Coping Skills Inventory-28 (ACSI-28) in competitive athletes remain scarce. Understanding how this integrated intervention influences neural activity and psychological coping mechanisms may contribute to advancing sport psychology and the design of multidimensional mental training programs.

The aim of this study was to examine the effects of a combined breathing control and self-talk intervention on EEG parameters and the ACSI-28 in elite national-level athletes. We hypothesized that the combined intervention would produce measurable changes in EEG markers associated with psychological regulation (e.g., attention and anxiety modulation) and improvements in coping skills, which may contribute to enhanced psychological readiness for competition. Specifically, we anticipated alterations in theta [31] and beta [32] power. EEG signals were analyzed across six brain areas using corresponding electrodes, reflecting functional and anatomical brain organization [33, 34].

## Materials and methods

### Participants

**Fig. 1:**
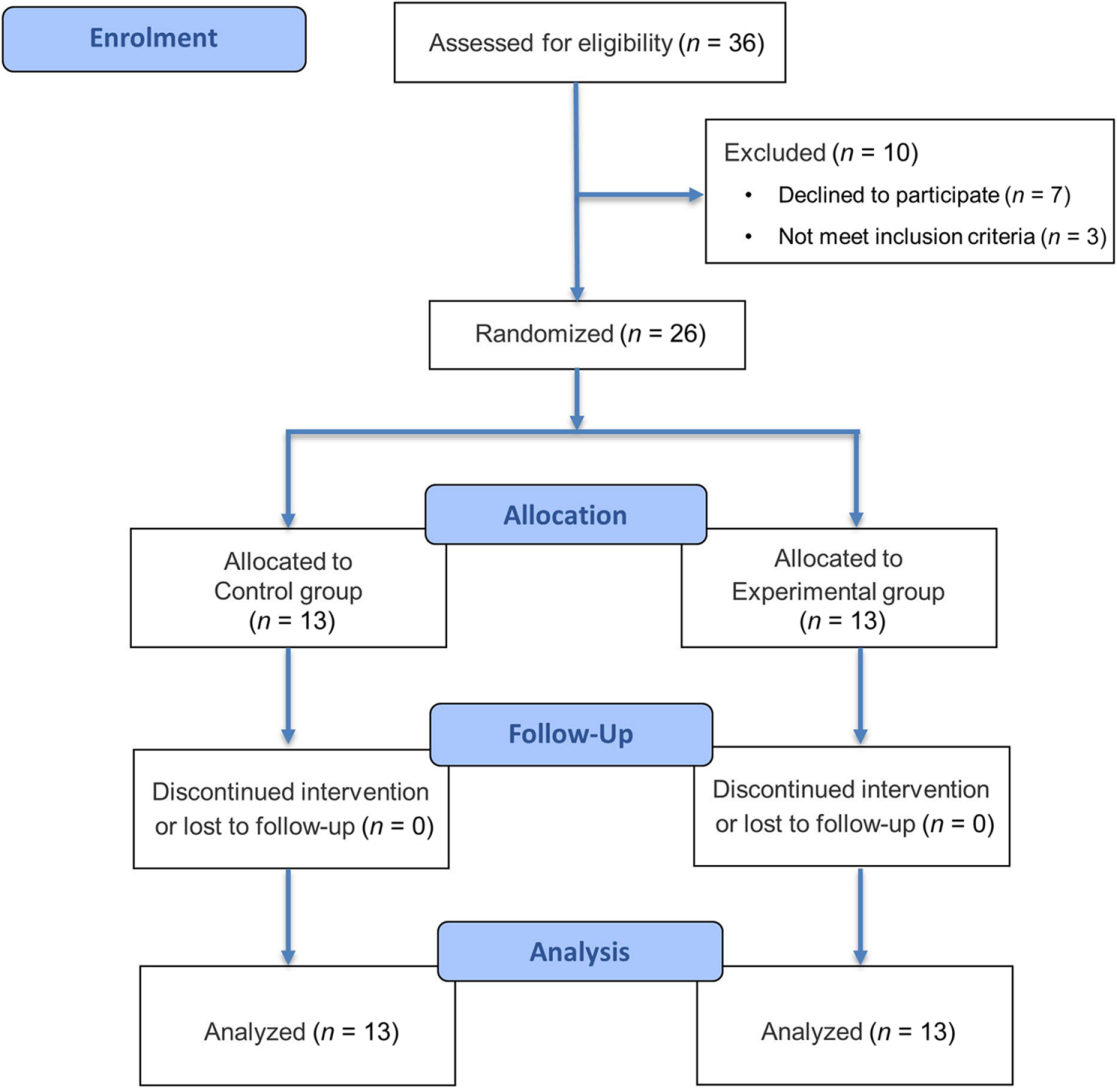
Participant recruitment flow chart

The primary interaction of interest (Condition x EEG response) in the previous EEG research [35] demonstrated a partial eta-squared $\:{\eta\:}_{\rho\:}^{2}$ = 0.110. This effect was converted to Cohen’s *\:f* using $\:f=\sqrt{{\eta\:}_{\rho\:}^{2}}$/(1- $\:{\eta\:}_{\rho\:}^{2}$), yielding *\:f* = 0.350. For a two-group comparison, this effect-size corresponds to approximately Cohen’s *d* = 0.700 ($\:d=2\:f)$. Using these effect-size estimates, an a priori power analysis was performed in G*power (α = 0.05, 1 – β = 0.80). For an independent two-sample test approximately 33 participants per group (*N* = 66) would be required. The present study recruited 13 participants per group (*N* = 26). Therefore, statistical power for detecting an effect of this magnitude is limited. Observed effect sizes (Cohen’s d) and 95% confidence intervals are reported to support transparent interpretation. Twenty-six elite national-level athletes (7 male, 19 females; mean±SD age: 20.9 ± 3.2 years; height: 166.4 ± 9.2 cm; weight: 58.0 ± 11.3 kg) volunteered for the study, consisting of eleven swimmers and fifteen gymnasts. Exclusion criteria included acute injury, a history of head injury, or neurological disease. Twenty-two participants were right-side dominant and four were left-side dominant. All participants were members of the Thai National Swimming and Gymnastic Teams and were preparing for the Southeast Asian Games, an international regional competition. These athletes train year-round in high-performance programs and have an average of 3.7 years (range 3–9 years) of competitive experience. Athletes were provided with detailed information regarding the study’s objectives, procedures, and potential risks, before providing written informed consent. Participants were randomized to either the experimental or control group. The CONSORT study flow diagram is presented in Fig. 1. Stratified random sampling by sex and sport type was first applied to ensure comparable proportions in both groups, followed by simple random sampling within each stratum. This experiment was approved and performed in accordance with the research ethics committee of Mahidol University - Central Institutional Review Board (COA No. MU-CIRB 2021/147.2206). It was also conducted in accordance with the Declaration of Helsinki.

### Procedure and data collection

This study investigated the effects of a combined breathing control and self-talk intervention on neural activity and coping skills in elite national-level athletes. Data were collected at two time points: baseline (pre-intervention) and post-intervention (one week prior to competition). The measurements included electroencephalogram (EEG) data and scores from the Athletic Coping Skills Inventory-28 (ACSI-28). At each measurement point, participants first completed the demographic questionnaire and ACSI-28, followed by having an EEG recording.

Resting-state EEG assessments were conducted in the morning for both pre- and post-intervention sessions to reduce circadian influences. Participants received standardized instructions the previous day regarding required sleep duration, restricted medication use, and rest level. Before each session, participants completed a brief readiness check covering sleep quality, fatigue, and overall physical state; testing was rescheduled if the participants were not adequately rested. To minimize variation in training load, the research team coordinated with coaching staff to ensure that training intensity and timing before pre- and post-intervention sessions were comparable. For female participants, menstrual cycle information was collected to avoid testing during periods when hormonal fluctuations may influence the EEG signal. These procedures were implemented to reduce potential confounding influences on the resting-state EEG recording. Data collection was conducted in a dedicated, environmentally-controlled (humidity and temperature) and quiet room at the athletes’ training camp. The same room was used for pre- and post-intervention measurements across both groups. EEG data were acquired while participants were seated in a relaxed, upright posture with their hands resting on their lap to minimize movement artifacts. Participants were explicitly instructed to maintain immobility and open eyes throughout the five-minute recording. To minimize the influence of cognitive processing and ensure a baseline resting-state measure, participants were directed to maintain a neutral mental state, characterized by passive observation without engaging in directed thought or task performance. A demonstration and explanation of the EEG equipment and procedures were provided to ensure participant comfort and familiarity before data collection commenced. All EEG recording and artifact evaluations were performed by a single experienced and trained examiner using a standardized protocol, thereby eliminating inter-rater variability. Consistency across sessions was ensured through fixed recording parameters, identical electrode placement procedures, impedance thresholds (< 20 kΩ), and controlled environmental conditions. Automated artifact rejection based on amplitude thresholds (± 150 µV) further reduced subjective variability. Following the baseline data collection, the sport psychologist explained the intervention and provided practice instructions to participants in the experimental group.

Adherence and fidelity were monitored throughout the 4-week program. The sport psychologist documented detail of each session, including attendance and duration. All participants in the experimental group completed 16/16 sessions (100% adherence), and all sessions followed the structured intervention protocol. In the control group, consultations were only provided when requested. One gymnast requested four sessions, and two swimmers requested one session each, resulting in a total of six sessions across the 13 control participants (mean 0.46 sessions per participant). These consultations were unstructured, involved goal-setting techniques, and covered general topics such as quality of life, training, and the weather.

### Blinding and analyst masking

Only the statistical analyst was blinded to group allocations and conducted all data analyses using anonymized datasets coded as group A and group B. Due to the nature of the intervention, it was not possible to blind the sport psychologist or participants to group allocation. Participants were aware of the type of sessions they received (combined self-talk and breathing control intervention vs. routine consultation), and therefore true participant blinding was highly difficult.

### Combined breathing control and self-talk intervention protocol

The intervention was designed to improve the athletes’ ability to cope with anxiety and develop relaxation skills. The combined intervention consisted of psychological techniques, consisting of breathing control and self-talk. The intervention structure adhered to the recommendation that psychological skill training should be performed for 15–30 min per session, 3–4 times per week [36]. Furthermore, slow-paced based breathing was incorporated due to its documented relaxing and anti-anxiety effects [37, 38]. For the experimental group, each intervention session was 15 min in duration, consisting of three 5-minute cycles of the combined breathing control and self-talk intervention. These sessions were performed four times per week (on Mondays, Wednesdays, Fridays, and Sundays) for a period of one month. A total of 16 sessions were administered for the intervention. The steps for combined breathing control and self-talk intervention were as follows:

1. The participants began by sitting in a chair with a backrest, maintaining a comfortable and relaxed posture for one minute.
2. Next, they focused their attention on their breath, becoming aware of the sensation of air entering and exiting through their nose for one minute.
3. This was followed by paced breathing, where they inhaled for a 3-second count and exhaled for a 4-second count. This step was performed for 2 min.
4. Then, participants were instructed to say the word “relax” during the exhalation phase for three cycles (30 s).
5. Finally, they were instructed to say the word “calm” during exhalation phase for three cycles (30 s).

### EEG and ACSI-28 measurements

The EEG instrument (eego™mylab, ANT Neuro, USA) consisted of 32 recording channels. These channels were grouped into six anatomical brain areas: Prefrontal (Fp_1_, Fp_z_, Fp_2_), Frontal (F_7_, F_3_, F_z_, F_4_, F_8_, FC_5_, FC_1_, FC_2_, FC_6_), Temporal (T_7_, and T_8_), Central (C_3_, C_z_, C_4_, CP_5_, CP_1_, CP_2_, CP_6_), Parietal (P_3_, P_7_, P_z_, P_4_, P_8_, PO_z_), Occipital (O_1_, O_z_, O_2_). CP_z_ served as the reference electrode, and GND as the ground electrode. Electrode impedances were maintained below 20 kΩ, following established recommendations [39]. All preprocessing steps and spectral analyses were performed using Advanced Source Analysis software (ASA, ANT Neuro, version 4.10.1). EEG signals were recorded at a sampling rate of 512 Hz and preprocessed following widely adopted EEG signal processing procedures [40–43]. Continuous data were band-pass filtered between 0.3 and 30 Hz using a zero-phase Butterworth filter to preserve the temporal integrity of the signal, followed by a 50 Hz notch filter to suppress power-line interference. Artifact detection was performed using an amplitude-based rejection criterion (± 150 µV), which was selected to remove gross artifacts, particularly ocular artifacts such as eye blinks that typically produce large voltage deflections in resting-state EEG recordings [42, 44]. Although more stringent thresholds are often used in event-related potential (ERP) studies (± 75–100 µV) [43], a slightly more liberal threshold is commonly adopted in resting-state spectral analyses to preserve sufficient data for reliable power estimation [41]. The same amplitude-based criterion was applied to exclude other non-physiological artifacts, including movement-related disturbances and high-amplitude EMG contamination. Additionally, slow drifts and high-frequency noise were attenuated via band-pass filtering (0.3–30 Hz), ensuring overall signal quality prior to spectral analysis [41, 44]. Although high-pass filtering attenuates DC components, an additional DC offset correction was applied to eliminate any residual baseline shifts and to ensure zero-mean signals prior to Fast Fourier Transformation (FFT) based spectral analysis, thereby improving numerical stability. Following artifact removal, the cleaned EEG signals were segmented into 2-second non-overlapping epochs for spectral analysis. Power spectral density (PSD) was computed using FFT implemented in ASA software. A Hanning window was applied to each epoch to reduce spectral leakage, resulting in a frequency resolution of approximately 0.5 Hz. PSD values were extracted for the standard frequency bands: delta (0.3–4 Hz), theta (4.5–8 Hz), alpha (8.5–13 Hz), and beta (13.5–30 Hz). No channels met the criteria for bad-channel detection; therefore, no channel interpolation procedures were required. Because the preprocessing pipeline employed automatic amplitude-based artifact rejection, the software does not provide a direct summary of the percentage of rejected epochs. Nevertheless, the rejection threshold and standardized preprocessing ensured consistent treatment of artifacts across participants.

The Thai version of the Athletic Coping Skills Inventory-28 (ACSI-28) was used to assess athletes’ coping skills. This version was previously validated in a large sample of Thai youth athletes, demonstrating acceptable internal consistency (overall α = 0.750) and strong construct validity (x^2^/df = 1.12, RMSEA = 0.018, CFI = 0.990, GFI = 0.930, AGFI = 0.920) while retaining the original seven-subscale structure [45]. The questionnaire consisted of 28 items grouped into seven subscales (four items each): (1) coping with adversity, (2) peaking under pressure, (3) goal setting/mental preparation, (4) concentration, (5) freedom from worry, (6) confidence, and achievement motivation (7) coachability. Participants rated each item on a 4-point Likert scale: 0 (almost never), 1 (sometimes), 2 (often), and 3 (almost always). Cronbach’s alpha coefficients were computed for the current sample to assess internal consistency.

### Statistical analysis

This study analyzed EEG data alongside the ACSI-28. Data normality was assessed using the Shapiro-Wilk test, and a subset of variables exhibited deviation from a normal distribution. Generalized linear mixed models (GLMM) were utilized to examine the linear relationships between groups, EEG across time points, and EEG across different brain areas. Although the raw variables exhibited deviation from normality, model diagnostics indicated that the residuals derived from the GLMM successfully approximated a normal distribution. This outcome, which validates the GLMM approach [46, 47], was verified through both visual inspection and formal testing (histograms, Q-Q plots, and Shapiro-Wilk p; see Supplementary File E). Consequently, a GLMM employing a gamma family distribution with a log link function was utilized for the analysis of the EEG data.

Repeated measures ANOVA were conducted to compare groups across the seven subscales of the ACSI-28. As the repeated measures factors possessed only two levels, sphericity was mathematically guaranteed (ε = 1), and Mauchly’s test was not applicable. The use of parametric models is supported by extensive evidence demonstrating that GLMM residual-based assumptions and repeated measures ANOVA are robust to moderate violations of normality. This robustness is particularly evident under balanced experimental designs and when appropriate corrections for sphericity or non-normality are implemented [48, 49]. When significant main effects were identified, post hoc analysis with Bonferroni correction was applied. Effect sizes (*d*) are reported to determine the magnitude of differences between sex groups and assessed against Cohen’s d definitions of: <0.2, 0.3, 0.5, and 0.8 for trivial, small, moderate, and large, respectively [50].

Finally, correlation analyses were performed to investigate the relationships between the PSD of specific EEG frequency bands and scores from the ACSI-28 questionnaire within each group. Spearman’s rank correlation coefficients were interpreted using the following thresholds: 0 to < 0.3 as negligible, 0.3 to 0.49 as low, 0.5 to 0.69 as moderate, and 0.7 to 0.89 as high, 0.9 to 1.0 as very high [51]. A Comprehensive set of 168 correlations was computed, derived from the cross-analysis of EEG band-power variables and ACSI-28 questionnaire (4 frequency bands x 6 brain regions x 7 ACSI-28 subscales). To mitigate the risk of Type I error inflation resulting from these multiple comparisons, p-values were adjusted using the false discovery rate (FDR) correction, specifically the Benjamini-Hochberg procedure. Statistical significance was uniformly defined as an FDR-adjusted *p* < .05. Statistical analyses were executed using the Jamovi software suite, supplemented by RStudio for specific computations. Data visualization was subsequently conducted using both RStudio and MATLAB. The results presented are restricted to findings demonstrating statistical significance (*p* < .05) or identifying critical interactions.

## Results

Baseline demographic, EEG, and pre-test ACSI- 28 data confirmed that the experimental and control groups were equivalent prior to the intervention (see Supplementary File A). The effects of the combined breathing control and self-talk intervention were subsequently examined by comparing the EEG and ACSI-28 data outcomes.

### EEG data in frequency bands

**Fig. 2:**
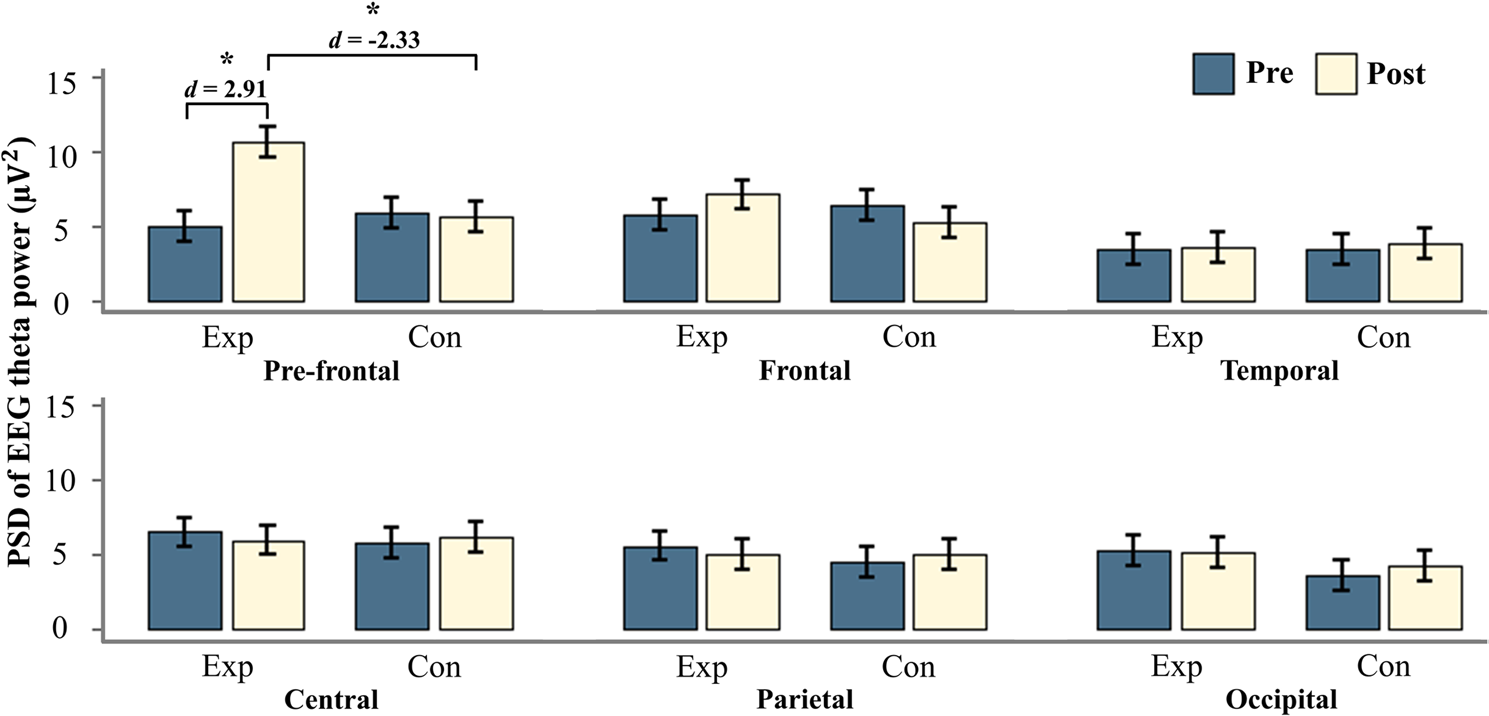
Estimated marginal means and 95% confidence interval of PSD for EEG theta power. Abbreviations: Exp = Experimental group, Con = Control group, *d* = Effect size (*d*_Cohen_). * Significance level set at *p* < .05

**Fig. 3:**
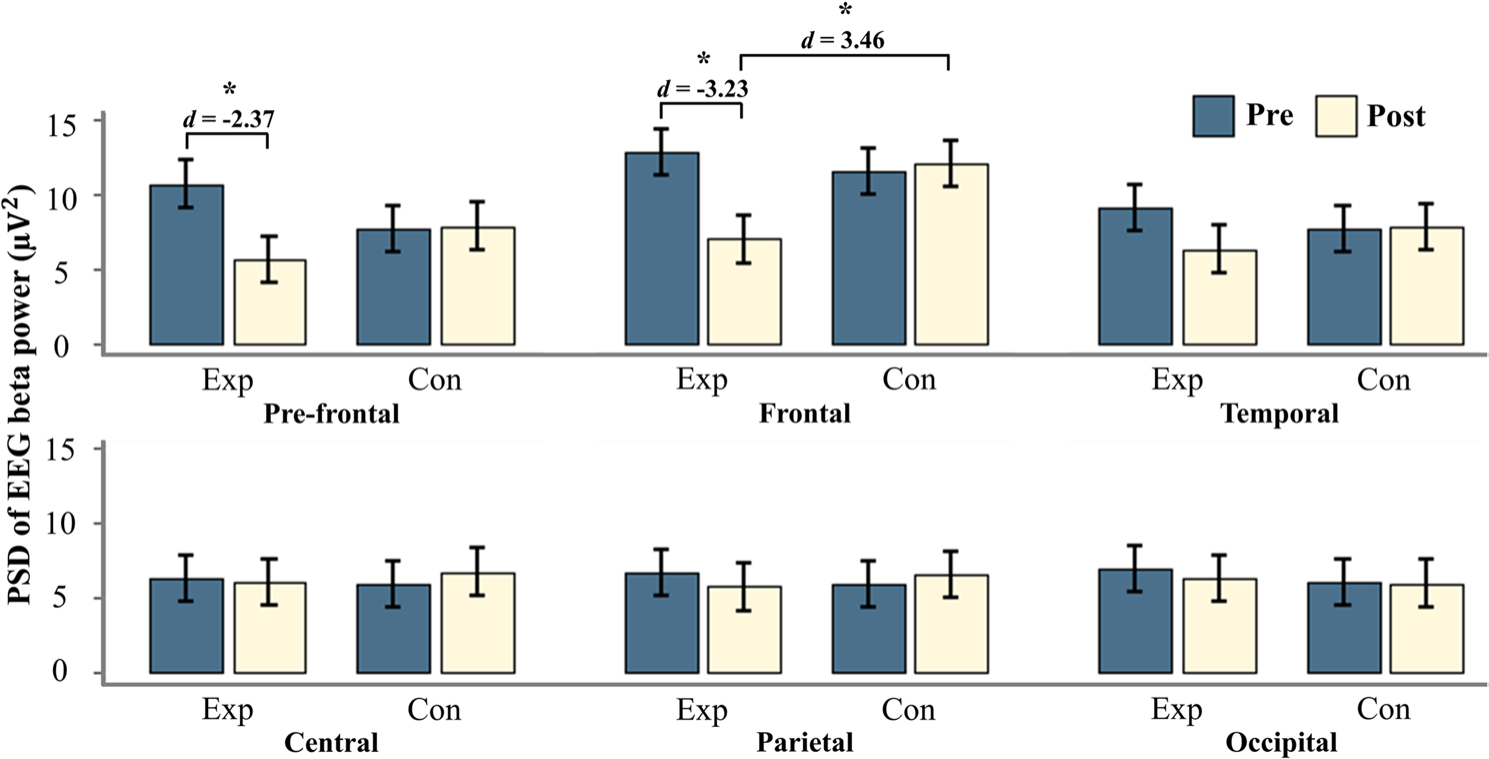
Estimated marginal means and 95% confidence interval of PSD for EEG beta power. Abbreviations: Exp = Experimental group, Con = Control group, *d* = Effect size (*d*_Cohen_). * Significance level set at *p* < .05

**Fig. 4:**
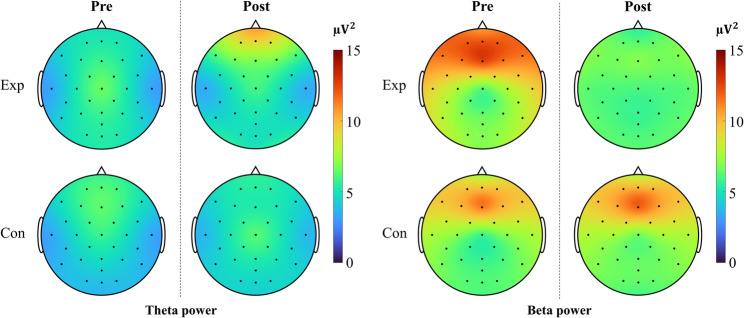
Grand average EEG topographic maps of PSD (µ$\:{\mathrm{V}}^{2}$) in the theta and beta power for the experimental and control groups

The EEG analysis revealed a significant three-way interaction among group, time, and brain area in the theta (*p* < .001) and beta (*p* = .048) power, as illustrated in Table 1. As presented in Table 1, the experimental group exhibited a significant increase in theta power within the prefrontal area compared to the control group at the post-intervention assessment (*p* < .001, mean difference = 5.04, *d* = -2.33, 95% CI: 3.62 to 6.46). Concurrently, beta power within the frontal area demonstrated a significant decrease in the experimental group relative to the control group at the same time point (*p* = .003, mean difference = -5.06, *d* = 3.46, 95% CI: -7.26 to -2.86). The estimated marginal means, including the mean and 95% confidence intervals (95% CI), are visually represented in Figs. 2 and 3, respectively. Furthermore, grand average EEG topographic maps illustrating power spectral density (PSD) in the theta and beta frequency bands at pre- and post-intervention periods are displayed in Fig. 4.

**Table 1:** Generalized linear mixed model (GLMM) of EEG in group, time, and brain area

Model results(Fixed EffectOmnibus tests)	Delta power					Theta power					Alpha power					Beta power				
	Diff	*d*	95%CI		*p*	Diff	*d*	95%CI		*p*	Diff	*d*	95%CI		*p*	Diff	*d*	95%CI		*p*
			LB	UB				LB	UB				LB	UB				LB	UB	
Group*Time*Brain area					.982					<.001*					1.00					.048*
Post Hoc Tests																				
E*Pre*PF – C*Pre*PF	10.0	-.156	-19.3	39.5	1.00	-.901	.501	-2.32	.516	1.00	-2.36	.253	-9.96	5.22	1.00	3.02	-.966	.816	5.22	1.00
E*Post*PF– C*Post*PF	-18.3	.215	-47.6	11.2	1.00	**5.04**	**-2.33**	**3.62**	**6.46**	<.001*	-3.07	.343	-10.7	4.52	1.00	-2.25	1.18	-4.45	-.046	1.00
E*Pre*PF – E*Post*PF	3.10	-.033	-26.3	32.5	1.00	**-5.73**	**2.91**	**-7.15**	**-4.31**	<.001*	.763	-.097	-6.83	8.35	1.00	**5.05**	**-2.37**	**2.85**	**7.25**	.003*
C*Pre*PF – C*Post*PF	-25.2	.463	-54.6	4.18	1.00	.212	-.105	-1.21	1.63	1.00	.062	-.010	-7.53	7.65	1.00	-.226	.075	-2.42	1.98	1.00
E*Pre*F – C*Pre*F	11.0	-.411	-18.4	40.4	1.00	-.686	.441	-2.10	0.74	1.00	-2.52	.291	-10.1	5.07	1.00	1.34	-.630	-.864	3.54	1.00
E*Post*F – C*Post*F	-14.8	.439	-44.2	14.6	1.00	1.83	-.980	.424	3.26	1.00	-1.48	.179	-9.07	6.11	1.00	**-5.06**	**3.46**	**-7.26**	**-2.86**	.003*
E*Pre*F – E*Post*F	8.22	-.242	-21.2	37.6	1.00	-1.36	.818	-2.78	.056	1.00	-.542	.069	-8.13	7.05	1.00	**5.86**	**-3.23**	**3.66**	**8.06**	<.001*
C*Pre*F – C*Post*F	-17.6	.665	-47.0	11.8	1.00	1.16	-.655	-.256	2.58	1.00	.495	-.053	-7.09	8.09	1.00	-.543	.284	-2.74	1.66	1.00
E*Pre*T – C*Pre*T	19.0	-.620	-10.5	48.3	1.00	-.020	.020	-1.44	1.40	1.00	-1.17	.304	-8.76	6.42	1.00	1.43	-.278	-.774	3.63	1.00
E*Post*T – C*Post*T	-11.4	.399	-40.8	18.0	1.00	-.267	.156	-1.69	1.15	1.00	-.684	.194	-8.27	6.91	1.00	-1.52	.405	-3.72	.684	1.00
E*Pre*T – E*Post*T	17.1	-.496	-12.3	46.5	1.00	-.148	.092	-1.57	1.27	1.00	-.127	.037	-7.72	7.46	1.00	2.81	-.617	.616	5.02	1.00
C*Pre*T – C*Post*T	-13.2	.558	-42.6	16.2	1.00	-.396	.344	-1.82	1.02	1.00	.360	-.093	-7.23	7.95	1.00	-.129	.029	-2.33	2.07	1.00
E*Pre*C – C*Pre*C	9.59	-.495	-19.8	39.0	1.00	.739	-.507	-.676	2.16	1.00	-.939	.088	-8.53	6.65	1.00	-.401	-.184	-1.80	2.60	1.00
E*Post*C – C*Post*C	-6.17	.239	-35.5	23.3	1.00	-.214	.088	-1.64	1.20	1.00	.029	-.011	-7.56	7.62	1.00	-.713	.315	-2.91	1.49	1.00
E*Pre*C – E*Post*C	6.47	-.261	-23.0	35.8	1.00	.515	-.214	-.896	1.94	1.00	-.690	.067	-8.28	6.90	1.00	.255	-.108	-1.95	2.45	1.00
C*Pre*C – C*Post*C	-9.29	.449	-38.7	20.1	1.00	-.438	.281	-1.86	.976	1.00	.278	-.033	-7.31	7.87	1.00	-.860	.407	-3.06	1.34	1.00
E*Pre*P – C*Pre*P	8.22	-.415	-21.2	37.6	1.00	1.07	-.829	-.336	2.50	1.00	1.62	-.121	-5.97	9.21	1.00	.734	-.314	-1.47	2.93	1.00
E*Post*P – C*Post*P	-4.12	.181	-33.5	25.3	1.00	.015	-.004	-1.41	1.43	1.00	.899	-.081	-6.69	8.49	1.00	-.826	.359	-3.03	1.37	1.00
E*Pre*P – E*Post*P	7.24	-.304	-22.2	36.6	1.00	.546	-.246	-.866	1.97	1.00	-.346	.021	-7.94	7.24	1.00	.920	-.384	-1.28	3.12	1.00
C*Pre*P – C*Post*P	-5.10	.275	-34.5	24.3	1.00	-.511	.391	-1.94	.896	1.00	-1.07	.100	-8.66	6.52	1.00	-.640	.287	-2.84	1.56	1.00
E*Pre*O – C*Pre*O	15.4	-.756	-14.0	44.8	1.00	1.62	-1.10	.194	3.03	1.00	2.18	-.144	-5.41	9.77	1.00	.871	-.275	-1.33	3.07	1.00
E*Post*O – C*Post*O	1.80	-.079	-27.6	31.2	1.00	.912	-.386	-.496	2.34	1.00	1.09	-.111	-6.50	8.68	1.00	.292	-.101	-1.91	2.49	1.00
E*Pre*O – E*Post*O	6.13	-.222	-23.3	35.5	1.00	.058	-.02	-1.37	1.47	1.00	1.57	-.095	-6.02	9.16	1.00	.679	-.192	-1.52	2.88	1.00
C*Pre*O – C*Post*O	-7.50	.557	-36.9	21.9	1.00	-.646	.525	-2.06	.776	1.00	.487	-.045	-7.11	8.07	1.00	.101	-.042	-2.10	2.30	1.00

*Abbreviations*: *E* Experimental group, *C* Control group, *Pre* Recording data at 3 months (pre-intervention), *Post* Recording data at 1 week (post-intervention), *95%CI LB* 95% Confidence interval lower bound, *95%CI UB* 95% Confidence interval upper bound, *d* Effect size (dCohen), *Diff* Mean difference, *PF* Pre-frontal area, *F* Frontal area, *T* Temporal area, *C* Central area, *P* Parietal area, *O* Occipital area

* Significance level seat at *p* < 0.05

### Subscales of the ACSI-28 questionnaire data

In the current sample (*N* = 26), pre-intervention alphas were exceptionally high (α = 1.00), reflecting highly restricted variability in baseline responses among elite athletes. Post-intervention alphas ranged from 0.581 to 0.840 across subscales, with good reliability for the total scale (α = 0.826) as shown in Table 2. The relatively lower reliability of coachability (α = 0.432) is acknowledged as a limitation

**Table 2:** Internal consistency (Cronbach’s alpha) for ACSI-28 at pre- and post-intervention

ACSI-28 subscale	Pre-intervention α	Post-intervention α
Coping with adversity	1.00	0.840
Coachability	1.00	0.432
Concentration	1.00	0.583
Confidence and achievement motivation	1.00	0.581
Goal setting/Mental preparation	1.00	0.689
Peaking under pressure	1.00	0.835
Freedom from worry	1.00	0.602
Total ACSI-28 subscale	**1.00**	**0.826**

*Abbreviations*: *ACSI-28 * Athletic Coping Skills Inventory-28

**Fig. 5:**
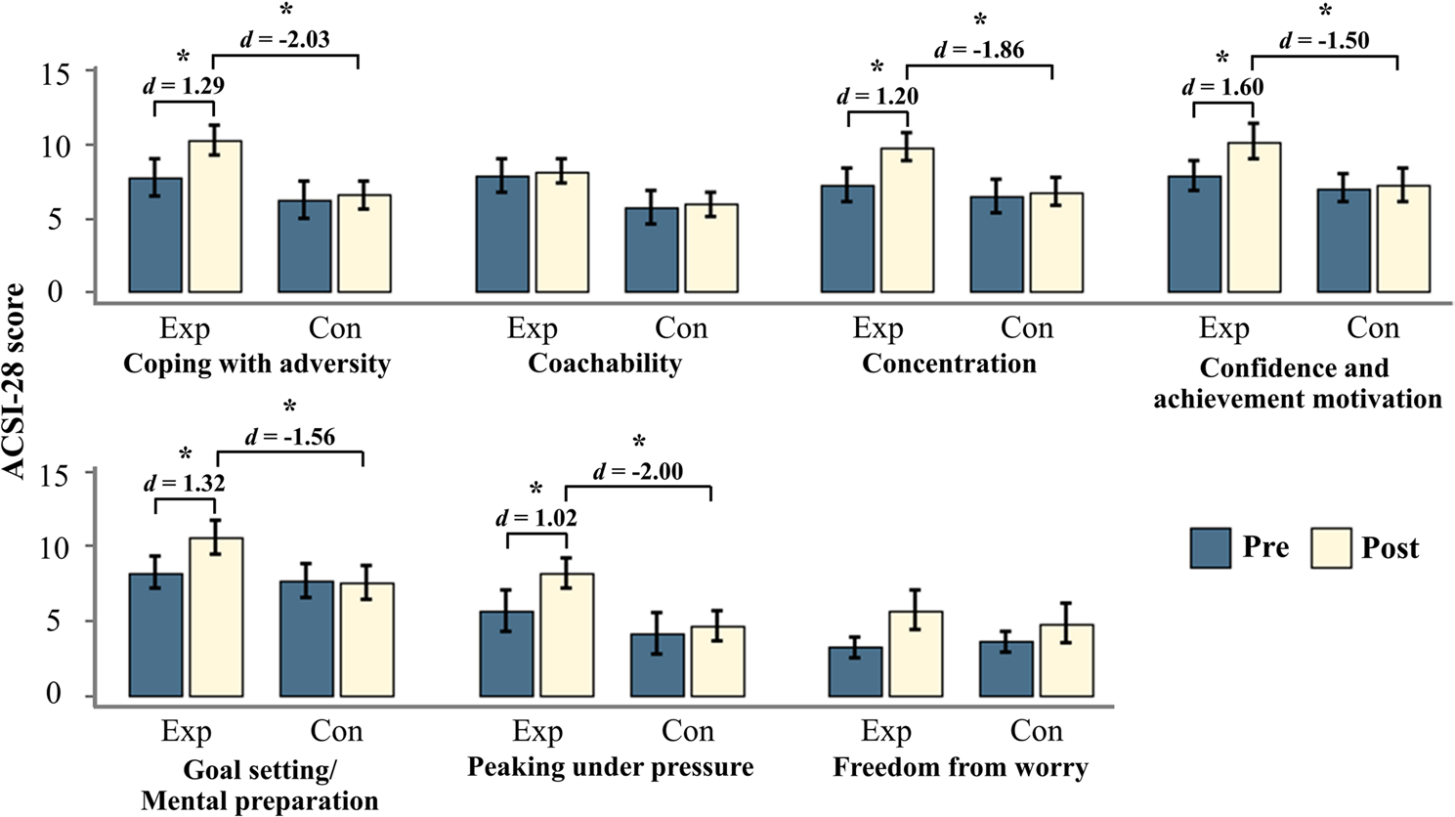
Estimated marginal means and 95% confidence interval of ACSI-28 subscales. Abbreviations: Exp = Experimental group, Con = Control group, d = Effect size (dCohen). * Significance level set at *p* < 0.05

As presented in Table 3, the repeated measures ANOVA revealed significant group-by-time interaction effects across several subscales. Significant differences between the experimental and control groups were observed in coping with adversity (*p* = .018, = 0.212), concentration (*p* = .027, = 0.188), confidence and achievement motivation (*p* = .039, = 0.166), goal setting/mental preparation (*p* = .009, = 0.251), and peaking under pressure (*p* = .043, = 0.160). Post hoc analysis conducted at the post-intervention time point (one week prior to the competition) demonstrated that the experimental group achieved significantly higher scores than the control group in coping with adversity (*p* < .001, mean difference = 3.69, *d* = -2.03, 95% CI: 2.40 to 4.98), concentration (*p* < .001, mean difference = 3.00, *d* = -1.86, 95% CI: 1.76 to 4.24), confidence and achievement motivation (*p* = .006, mean difference = 2.92, *d* = -1.50, 95% CI = 1.40 to 4.44), goal setting/mental preparation (*p* = .003, mean difference = 3.08, *d* = -1.56, 95% CI: 1.57 to 4.59), and peaking under pressure (*p* < .001, mean difference = 3.54, *d* = -2.00, 95% CI: 2.17 to 4.89). The estimated marginal means of the ACSI-28 data, including the mean and 95% confidence intervals (95% CI), are visually presented in Fig. 5

**Table 3:** Repeated measures ANOVA of ACSI-28 subscales in group and time

Subscales	Group*Time			Post Hoc Tests					
	**Mean square**	$\:{\boldsymbol{n}}_{\boldsymbol{p}}^{2}$	***p***	Comparisons - Group*Time	**Diff**	***d***	**95%CI**		**p**_**bonferroni**_
							**LB**	**UB**	
Coping with adversity	**15.1**	**0.212**	0.018*	E*Pre – C*Pre	1.54	− 0.699	− 0.154	3.23	0.526
				E*Post – C*Post	**3.69**	**-2.03**	**2.40**	**4.98**	< 0.001*
				E*Pre – E*Post	**-2.54**	**1.29**	**-4.04**	**-1.04**	0.002*
				C*Pre – C*Post	− 0.385	0.215	-1.89	1.11	1.00
Coachability	0.019	0.001	0.908	E*Pre – C*Pre	2.15	-1.14	0.702	3.62	0.032
				E*Post – C*Post	2.23	-1.51	1.09	3.37	0.005
				E*Pre – E*Post	− 0.308	0.172	-1.61	1.01	1.00
				C*Pre – C*Post	− 0.231	0.139	-1.54	1.08	1.00
Concentration	**16.2**	**0.188**	0.027*	E*Pre – C*Pre	0.769	− 0.378	− 0.796	2.34	1.00
				E*Post – C*Post	**3.00**	**-1.86**	**1.76**	**4.24**	< 0.001*
				E*Pre – E*Post	**-2.54**	**1.20**	**-3.95**	**-1.13**	0.005*
				C*Pre – C*Post	− 0.308	0.208	-1.72	1.10	1.00
Confidence and achievement motivation	**14.0**	**0.166**	0.039*	E*Pre – C*Pre	0.846	− 0.506	− 0.442	2.14	1.00
				E*Post – C*Post	**2.92**	**-1.50**	**1.40**	**4.44**	0.006*
				E*Pre – E*Post	**-2.31**	**1.60**	**-3.71**	**− 0.889**	0.013*
				C*Pre – C*Post	− 0.231	0.107	-1.64	1.18	1.00
Goal setting/Mental preparation	**20.9**	**0.251**	0.009*	E*Pre – C*Pre	0.538	− 0.287	− 0.907	1.99	1.00
				E*Post – C*Post	**3.08**	**-1.56**	**1.57**	**4.59**	0.003*
				E*Pre – E*Post	**-2.39**	**1.32**	**-3.87**	**− 0.912**	0.006*
				C*Pre – C*Post	0.154	− 0.074	-1.33	1.63	1.00
Peaking under pressure	**13.0**	**0.160**	0.043*	E*Pre – C*Pre	1.54	− 0.627	− 0.345	3.42	0.738
				E*Post – C*Post	**3.54**	**-2.00**	**2.17**	**4.89**	< 0.001*
				E*Pre – E*Post	**-2.46**	**1.02**	**-4.10**	**− 0.817**	0.006*
				C*Pre – C*Post	− 0.462	0.256	-2.11	1.17	1.00
Freedom from worry	4.92	0.068	0.199	E*Pre – C*Pre	− 0.385	0.305	-1.37	0.594	1.00
				E*Post – C*Post	0.846	− 0.369	− 0.912	2.59	1.00
				E*Pre – E*Post	-2.46	1.21	-3.88	-1.04	0.006
				C*Pre – C*Post	-1.23	0.750	-2.65	0.191	0.445

*Abbreviations*: *E* Experimental group, *C* Control group, *Pre* Recording data at 3 months (pre-intervention), *Post* Recording data at 1 week (post-intervention), *95%CI LB* 95% confidence interval lower bound, *95%CI UB* 95% confidence interval upper bound, *d* Effect size (dCohen), *Diff * Mean difference

* Significance level seat at *p* < .05

### Correlation between EEG power spectral density and ACSI-28

The correlation analyses investigated the association between the EEG power spectral density across frequency bands and the ACSI-28 subscale scores in both the experimental and control groups. In the experimental group, a significant negative correlation was observed only within the delta frequency band. Delta power in the prefrontal area was significantly associated with goal setting/mental preparation (Spearman’s rho = − 0.702, FDR-adjusted *p* = .042). Significant correlations were also found in the control group, spanning delta and theta frequency bands. Specifically, delta power in three areas comprising prefrontal (Spearman’s rho = − 0.851, FDR-adjusted *p* = .006), frontal (Spearman’s rho = − 0.669, FDR-adjusted *p* = .024), and central (Spearman’s rho = − 0.700, FDR-adjusted *p* = .024) were significantly negatively associated with coping with adversity. Furthermore, theta activity in the frontal area showed a significant positive correlation with confidence and achievement motivation (Spearman’s rho = 0.718, FDR-adjusted *p* = .036). The magnitude of these significant correlations ranged from moderate to high (0.669 to 0.851) in both the experimental and control groups.

## Discussion

The present study examined the effects of a combined breathing control and self-talk intervention on elite national-level athletes. We specifically examined changes in self-assessment of coping skills and electroencephalogram (EEG) by comparing an experimental group to a control group. Our findings suggest changes in both neural activity and self-assessed psychological skills following the combined intervention, indicating that this combined intervention may influence neurophysiological markers related to processes such as anxiety regulation and attention, as well as self-reported coping skills. Consistent with our hypothesis, the combined intervention was associated with changes in EEG activity. We observed an increase in theta power in the prefrontal cortex and a decrease in beta power in the frontal cortex when compared to the control group. These neural findings occurred alongside potential improvements in self-assessed psychological skills. However, the relationship between these neural and subjective changes remains unclear. Specifically, the experimental group showed an increase in several ACSI-28 subscales, including coping with adversity, concentration, confidence and achievement motivation, goal setting/mental preparation and peaking under pressure.

We interpreted our findings based on the power spectral density (PSD) values, which are typically expressed in microvolts squared ($\:{\mathrm{V}}^{2}$), representing the signal power contained within each respective frequency band [52]. The observed magnitude of these differences falls within the neurophysiologically meaningful range reported in previous EEG studies examining cognitive and psychophysiological training in athletes [53, 54], suggesting that the measured changes may reflect a functionally relevant modulation of cortical activity [55]. However, EEG measures are inherently variable and may be influenced by individual and contextual factors. The observed decrease in beta power in the frontal cortex in our experimental group may be interpreted as being consistent with changes in attention and sensory processing, a finding that aligns with previous research [56]. Beta power has been associated with neurocognitive functions and network dynamics, including attentional orienting [57], cognitive effort [58], and working memory [59]. Therefore, the changes we observed in beta power could correspond to alterations in the activity of cognitive networks that operate across a variety of tasks [32]. Furthermore, the observed decrease in beta power may be associated with reduced arousal or anxiety-related processes [60], which are often linked to the relaxation effects of breathing interventions [61]. However, such interpretations should be made cautiously, given the indirect nature of resting-state EEG measures.

Furthermore, the combined intervention produced an increase in prefrontal theta power in the experimental group relative to the control group. Theta oscillations in the prefrontal cortex have been linked to several processes relevant to anxiety and cognitive control, including threat detection, conflict monitoring, and top-down emotion regulation [62–66]. The literature on theta-anxiety relationships is not uniform; while some studies report that increases in theta power emerge during anxious or uncertain states [64], whereas others associate increased theta power with a sense of safety [65]. These divergent findings suggest that prefrontal theta oscillations are context-dependent and likely reflect a combination of vigilance, regulatory engagement, and cognitive control processes. In our study, the observed theta power increase may be consistent with increased engagement of regulatory processes, rather than necessarily reflecting heightened anxiety. This interpretation is supported by the fact that theta power is also associated with control and response inhibition [66], functions that are typically engaged during regulatory strategies such as controlled breathing control and self-talk.

When considered alongside the observed reduction in frontal beta power, the overall pattern is more consistent with improved regulatory efficiency and attentional control rather than elevated threat processing. Thus, while theta power changes alone cannot be interpreted as a direct marker of reduced or increased anxiety, the combined pattern of increased theta and decreased beta power is consistent with a pattern that may reflect more efficient top-down regulatory engagement.

Breathing-related entrainment is known to occur in cortical neural networks, which are critical for psychological factors including emotion, cognition and memory [67, 68]. Interventions involving breathing have been shown to produce consistent anti-arousal changes in affective states [69] and to reduce feelings of stress and anxiety during exercise [70]. Our findings are consistent with the previous study by Cheng et al., which demonstrated that 0.1 Hz breathing can alter EEG [71]. Specifically, it can increase frontal theta power, and this change is interpreted as being associated with enhanced attentional engagement [72]. Additionally, self-talk is a well-established technique that enhances top-down control, a process synonymous with self-control. It has been shown to improve awareness and problem-solving skills, and increased resilience [11, 73]. These benefits are also reflected in applied settings, such as improved sport performance, and also enhance cognitive functions [74]. Our findings suggest that the intentional self-talk may contribute to changes in neural patterns that are often associated with more regulated or focused mental states, rather than directly indicating a transition between specific psychological states. This is evidenced by the balanced mix of beta and theta power observed in our EEG data. These changes in neural activity may be related to the observed improvements in psychological coping skills following the combined breathing control and self-talk intervention. However, this relationship remains inferential and is not directly demonstrated in the present design.

Regarding the Athletic Coping Skills Inventory-28 (ACSI-28) questionnaire results, we found a post-intervention increase in five subscales for the experimental group compared to the control group: coping with adversity, concentration, confidence and achievement motivation, goal setting/mental preparation, and peaking under pressure. These findings suggest that the 4-week combined intervention provides preliminary evidence that the intervention may contribute to improvements in these psychological skills. The observed increase in the goal setting/mental preparation subscale may be linked to the self-talk component of the intervention. The ‘relax and calm’ self-talk used in the study may serve as a form of mental rehearsal, allowing athletes to internalize and focus the situation [75]. This type of preparatory self-talk could help athletes to redirect their attention away from negative or anxiety-provoking thoughts. The mechanism by which these psychological scores were enhanced may be due to the influence of self-talk on emotional and cognitive control [76], which is further supported by the observed increase in the concentration and coping with adversity subscale scores. Furthermore, the breathing control component of the intervention may also contribute to the increased concentration subscale score, as controlled breathing is a well-established technique used to improve focus and maintain attention in athletes. This aligns with the proposed mechanism that breathing control can alter cognitive and emotional states [69] and may contribute to improved concentration. The increased scores in the confidence and achievement motivation and peaking under pressure subscale may be an indirect effect from the combined intervention. This might be mediated by changes in psychological processes such as anxiety regulation. However, EEG findings should be interpreted as indirect neural correlates rather than direct evidence of reduced anxiety. When athletes are better able to manage their anxiety, they typically experience an increase in confidence and a greater ability to perform effectively during high-pressure situations [17]. Overall, these findings collectively suggest that combined intervention may contribute to improvements in athletes’ coping skills. However, because participants knew the general nature of the sessions they received, expectancy effects or demand characteristics may have influenced self-reported measures such as ACSI-28. These factors are inherent challenges in psychological intervention research and should be considered when interpreting the findings.

The examination of associations between EEG activity and psychological skills revealed that only a selective set of correlations remained significant after applying false discovery rate (FDR) correction, indicating that the most robust EEG-ACSI-28 relationships were focused within specific frequency bands and frontal brain areas. In the experimental group, the only significant association was a negative relationship between prefrontal delta power and goal setting/mental preparation. Reduced delta activity in frontal areas is commonly interpreted as reflecting greater cortical activation and regulatory engagement [77, 78]. Thus, athletes who demonstrated strong cognitive preparation skills also exhibited a profile characterized by reduced delta activity (an activated prefrontal profile) during rest. This finding aligns with the intervention’s aim to enhance psychological readiness and self-regulatory control. A broader pattern emerged in the control group, where significant negative correlations linked delta power across prefrontal, frontal, and central areas with coping with adversity. These results suggest that athletes with superior adversity-coping abilities tend to show reduced resting state delta power, pointing to a more aroused or engaged cortical state. This aligns with recent EEG research linking low-frequency power suppression to effective emotion regulation and stress resilience [79]. A significant positive association between frontal theta power and confidence/achievement motivation was also observed. Frontal theta power is repeatedly linked to motivational drive, cognitive effort and allocation of attentional resources [66, 80].

Collectively, these findings highlight the potential role of frontal delta and theta activity in profiling psychological skills related to planning, coping, and motivation. The selective nature of the results after correction underscore the importance of interpreting EEG-behavior correlations cautiously and reinforces the idea that only the strongest and most theoretically meaningful neural-psychological associations should be emphasized [81]. Notably, the limited number of significant associations after multiple-comparison correction further suggest that neural and self-reported measures may operate partially independently. As such, any apparent convergence between EEG and psychological findings in the present study should be considered inferential rather than directly demonstrated.

Despite some incongruence between neural and self-assessment findings, our results suggest that the 4-week combined intervention was associated with improvements in athletes’ psychological skills at both neural and subjective levels. Specifically, EEG findings suggest potential changes in neural processes. However, these neural patterns do not directly correspond to self-reported psychological measures and may reflect distinct aspects of adaptation. These findings should be interpreted cautiously, particularly when linking neural activity to psychological processes. Overall, these findings indicate that the combined intervention may represent a promising approach for enhancing athletes’ psychological skills, with potential benefits for anxiety management and coping abilities in high-pressure situations. Importantly, given the use of resting-state EEG and the absence of objective behavioral performance measures, the present study findings should be interpreted as reflecting indirect neural correlates rather than specific markers of psychological states. The relationship between EEG frequency bands and constructs such as anxiety and attention remains complex and context-dependent, and therefore requires cautious interpretation. Given the relatively small sample size and study design, the present findings should be considered preliminary and exploratory in nature.

Our study has several limitations related to the research design and logistical constraints. First, because we were unable to include single-component control groups (breathing-only or self-talk-only), the design does not allow the unique contribution of each technique to be isolated. Therefore, the present study design does not allow causal inference regarding the independent contributions of breathing control and self-talk components of the combined intervention, and observed effects should be interpreted as reflecting the combined intervention rather than specific mechanisms. Furthermore, while control participants received only unstructured, on-request consultations, these interactions may still have produced non-specific benefits or placebo-like effects, as attention-control activities have been shown to influence outcomes in previous behavioral research [82], which limits the ability to attribute observed effects specifically to the intervention. However, because participants knew the general nature of the sessions they received, expectancy effects or demand characteristics may have influenced self-reported outcomes. Additionally, participants were not blinded to group allocation, which may have introduced expectancy or performance-related biases.

A second limitation is the restricted statistical power due to small sample size (*N* = 26). The relatively large effect sizes should be interpreted with caution, as they may reflect sample-specific characteristics or potential overestimation due to small sample size. Replication in larger samples is warranted. Although a priori calculations indicated that a larger sample would have been ideal, recruitment was constrained by the logistical demands of working with elite athletes during their competition preparation period. As a result, small-to-moderate effects may not have been reliably detected; however, reporting observed effect sizes and confidence intervals provides useful preliminary evidence to guide future research. This constraint also affected ACSI-28 subscales which demonstrated low internal consistency, likely due to the small sample size and limited score variability in this elite national-level athlete cohort. Therefore, the subscale-level findings offer useful preliminary insight that can guide subsequent psychometric validation efforts in elite athlete cohorts.

Third, data collection could not be conducted during actual competitions due to coaching and scheduling constraints, and no task or simulation replicating high-pressure competitive environments was used. While our resting-state EEG measurements reveal changes in neural markers associated with psychological states, these cannot be directly equated with improvements in objective performance ability. Confirming objective performance enhancement would require additional measures, such as task-based EEG, sport-specific motor performance tests, or induced pressure scenarios. Incorporating these conditions would have provided more ecologically valid insight into athletes’ neural and psychological response to stress and performance. Finally, generalizability is limited as the sample consisted exclusively of elite Thai national team athletes preparing for an international event. Therefore, the findings may not generalize to recreational athletes, developmental athletes, or competitors from other sports, age groups, or performance levels. Future studies should consider incorporating component-specific control groups, larger and more diverse samples, and ecologically valid performance simulations (e.g., virtual reality) to strengthen generalizability and interpretability.

## Conclusion

This study suggested that a combined breathing control and self-talk intervention may be an effective approach for enhancing psychological coping skills in elite national-level athletes. EEG analysis showed increased theta power in the prefrontal cortex and decreased beta power in the frontal cortex in the experimental group relative to the control group, patterns that may be associated with neural processes related to attentional and stress regulation. In addition, ACSI-28 scores increased across several subscales, including coping with adversity, concentration, confidence and achievement motivation, goal setting/mental preparation, and peaking under pressure. Taken together, these findings suggest that the combined intervention may contribute to improvements in aspects of mental resilience and performance under stress in this athletic population. However, these conclusions should be interpreted within the context of the present sample, and broader applications beyond the domain of sports warrant further investigation in larger and more diverse populations.

## Supplementary Information

Supplementary Material 1.

## Data Availability

The datasets generated and analyzed during this study are available from the corresponding author on reasonable request.

## Abbreviations

ACSI-28: Athletic Coping Skills Inventory-28
EEG: Electroencephalogram
FFT: Fast Fourier Transformation
FDR: False Discovery Rate
GLMM: Generalized Linear Mixed Modelling
GND: Ground Electrode
PSD: Power spectral density

## References

[1] Cashmore E. Sport and exercise psychology: the key concepts / Ellis Cashmore. Revised edition. ed. Milton Park, Oxfordshire: Milton Park, Oxfordshire : Routledge, 2008; 2008.

[2] Argiris ET, Mavvidis A, Tsigilis N. Dealing with stress during tennis competition. The association of approach-and avoidance-coping with metacognition and achievement goal theory perspectives. J Phys Educ Sport. 2018;18(4):2454–65.

[3] Lazarus RS. How emotions influence performance in competitive sports. sport Psychol. 2000;14(3):229–52.

[4] Lazarus RS, Folkman S. Stress, appraisal, and coping. New York: Springer publishing company; 1984.

[5] Benchehida A, Belmerabet F, Benchehida K, Why mental preparation is so important on directing of athletic performance? Eur J Phys Educ Sport Sci. 2016;2(5):140–52.

[6] Voss M, Kramer A, Basak C, Prakash R, Roberts B. Are Expert Athletes ‘Expert’ in the Cognitive Laboratory? A Meta-Analytic Review of Cognition and Sport Expertise. Appl Cogn Psychol. 2010;24:812–26.

[7] Williams A, Ford P. Expertise and expert performance in sport. Int Rev Sport Exerc Psychol. 2008;1:4–18.

[8] Chen J, Zhou D, Gong D, Wu S, Chen W. A study on the impact of systematic desensitization training on competitive anxiety among Latin dance athletes. Front Psychol. 2024;15:1371501. 38655213 10.3389/fpsyg.2024.1371501 PMC11037396

[9] Ohuruogu B, Jonathan UI, Ikechukwu UJ. Psychological Preparation for Peak Performance in Sports Competition. J Educ Pract. 2016;7(12):47–50.

[10] Bentley TGK, D’Andrea-Penna G, Rakic M, Arce N, LaFaille M, Berman R, et al. Breathing practices for stress and anxiety reduction: conceptual framework of implementation guidelines based on a systematic review of the published literature. Brain Sci. 2023;13(12):1612. 10.3390/brainsci13121612 PMC10741869 38137060

[11] Hatzigeorgiadis A, Zourbanos N, Galanis E, Theodorakis Y. Self-Talk and Sports Performance: A Meta-Analysis. Perspect Psychol Sci. 2011;6(4):348–56. 26167788 10.1177/1745691611413136

[12] de Matos LF, Bertollo M, Stefanello JMF, Pires FO, da Silva CK, Nakamura FY, et al. Motivational self-talk improves time-trial swimming endurance performance in amateur triathletes. Int J Sport Exerc Psychol. 2021;19(3):446–59.

[13] Cece V, Guillet Descas E, Martinent G. Mental training program in racket sports: a systematic review. Int J Racket Sports Sci. 2020;2(1):55–71.

[14] Hidayat Y, Budiman D. The influence of self-talk on learning achievement and self confidence. Asian Social Sci. 2014;10(5):186.

[15] Chang Y-K, Ho L-A, Lu FJ-H, Ou C-C, Song T-F, Gill DL. Self-talk and softball performance: The role of self-talk nature, motor task characteristics, and self-efficacy in novice softball players. Psychol Sport Exerc. 2014;15(1):139–45.

[16] Zetou E, Nikolaos V, Evaggelos B. The effect of instructional self-talk on performance and learning the backstroke of young swimmers and on the perceived functions of it. J Phys Educ Sport. 2014;14(1):27.

[17] Hatzigeorgiadis A, Zourbanos N, Mpoumpaki S, Theodorakis Y. Mechanisms underlying the self-talk–performance relationship: The effects of motivational self-talk on self-confidence and anxiety. Psychol Sport Exerc. 2009;10(1):186–92.

[18] Papaioannou A, Theodorakis Y, Ballon F, Auwelle YV. Combined effect of goal setting and self-talk in performance of a soccer-shooting task. Percept Mot Skills. 2004;98(1):89–99. 15058871 10.2466/pms.98.1.89-99

[19] Fritsch J, Feil K, Jekauc D, Latinjak AT, Hatzigeorgiadis A. The relationship between self-talk and affective processes in sports: A scoping review. Int Rev Sport Exerc Psychol. 2024;17(1):482–515.

[20] Hardy J. Speaking clearly: A critical review of the self-talk literature. Psychol Sport Exerc. 2006;7(1):81–97.

[21] Zaccaro A, Piarulli A, Laurino M, Garbella E, Menicucci D, Neri B, et al. How Breath-Control Can Change Your Life: A Systematic Review on Psycho-Physiological Correlates of Slow Breathing. Front Hum Neurosci. 2018;12:353. 30245619 10.3389/fnhum.2018.00353 PMC6137615

[22] Laborde S, Mosley E, Thayer JF. Heart Rate Variability and Cardiac Vagal Tone in Psychophysiological Research - Recommendations for Experiment Planning, Data Analysis, and Data Reporting. Front Psychol. 2017;8:213. 28265249 10.3389/fpsyg.2017.00213 PMC5316555

[23] Ma X, Yue ZQ, Gong ZQ, Zhang H, Duan NY, Shi YT, et al. The Effect of Diaphragmatic Breathing on Attention, Negative Affect and Stress in Healthy Adults. Front Psychol. 2017;8:874. 28626434 10.3389/fpsyg.2017.00874 PMC5455070

[24] Paul M, Garg K. The effect of heart rate variability biofeedback on performance psychology of basketball players. Appl Psychophysiol Biofeedback. 2012;37(2):131–44. 22402913 10.1007/s10484-012-9185-2

[25] Steffen PR, Bartlett D, Channell RM, Jackman K, Cressman M, Bills J, et al. Integrating Breathing Techniques Into Psychotherapy to Improve HRV: Which Approach Is Best? Front Psychol. 2021;12:624254. 33658964 10.3389/fpsyg.2021.624254 PMC7917055

[26] Kumar GP, Adarsh A, Ramakrishnan AG. Modulation of EEG by Slow-Symmetric Breathing Incorporating Breath-Hold. IEEE Trans Biomed Eng. 2025;72(4):1387–96. 40030340 10.1109/TBME.2024.3505963

[27] Bahi C, Irrmischer M, Franken K, Fejer G, Schlenker A, Deijen JB, et al. Effects of conscious connected breathing on cortical brain activity, mood and state of consciousness in healthy adults. Curr Psychol. 2024;43(12):10578–89.

[28] Goheen J, Anderson JAE, Zhang J, Northoff G. From Lung to Brain: Respiration Modulates Neural and Mental Activity. Neurosci Bull. 2023;39(10):1577–90. 37285017 10.1007/s12264-023-01070-5 PMC10533478

[29] Ribeiro LJA, Bastos V, Coertjens M. Breath-holding as model for the evaluation of EEG signal during respiratory distress. Eur J Appl Physiol. 2024;124(3):753–60. 38105311 10.1007/s00421-023-05379-x

[30] Anusha SA, Kumar GP, Ramakrishnan AG. Brain-scale theta band functional connectome as signature of slow breathing and breath-hold phases. Comput Biol Med. 2025;184:109435. 10.1016/j.compbiomed.2024.109435 39616883

[31] Ng HH, Wu CW, Huang FY, Cheng YT, Guu SF, Huang CM, et al. Mindfulness Training Associated With Resting-State Electroencephalograms Dynamics in Novice Practitioners via Mindful Breathing and Body-Scan. Front Psychol. 2021;12:748584. 34777144 10.3389/fpsyg.2021.748584 PMC8581621

[32] Skwara AC, King BG, Zanesco AP, Saron CD. Shifting Baselines: Longitudinal Reductions in EEG Beta Band Power Characterize Resting Brain Activity with Intensive Meditation. Mindfulness (N Y). 2022;13(10):2488–506. 36258902 10.1007/s12671-022-01974-9 PMC9568471

[33] Fan X, Zhou Q, Liu Z, Xie F. Electroencephalogram assessment of mental fatigue in visual search. Biomed Mater Eng. 2015;26(Suppl 1):S1455–63. 26405908 10.3233/BME-151444

[34] Strijkstra AM, Beersma DG, Drayer B, Halbesma N, Daan S. Subjective sleepiness correlates negatively with global alpha (8–12 Hz) and positively with central frontal theta (4–8 Hz) frequencies in the human resting awake electroencephalogram. Neurosci Lett. 2003;340(1):17–20. 12648748 10.1016/s0304-3940(03)00033-8

[35] Bing-Canar H, Pizzuto J, Compton RJ. Mindfulness‐of‐breathing exercise modulates EEG alpha activity during cognitive performance. Psychophysiology. 2016;53(9):1366–76. 27245493 10.1111/psyp.12678

[36] Gill DL, Williams L, Reifsteck EJ. Psychological dynamics of sport and exercise. 4th ed. Champaign: Human Kinetics; 2017.

[37] Van Diest I, Verstappen K, Aubert AE, Widjaja D, Vansteenwegen D, Vlemincx E. Inhalation/exhalation ratio modulates the effect of slow breathing on heart rate variability and relaxation. Appl Psychophysiol Biofeedback. 2014;39(3):171–80. 25156003 10.1007/s10484-014-9253-x

[38] Wells R, Outhred T, Heathers JA, Quintana DS, Kemp AH. Matter over mind: a randomised-controlled trial of single-session biofeedback training on performance anxiety and heart rate variability in musicians. 2012. 10.1371/journal.pone.0046597 PMC3464298 23056361

[39] Yokoyama H, Kaneko N, Masugi Y, Ogawa T, Watanabe K, Nakazawa K. Gait-phase‐dependent and gait‐phase‐independent cortical activity across multiple regions involved in voluntary gait modifications in humans. Eur J Neurosci. 2021;54(12):8092–105. 32557966 10.1111/ejn.14867

[40] Barry RJ, Clarke AR, Johnstone SJ, Magee CA, Rushby JA. EEG differences between eyes-closed and eyes-open resting conditions. Clin Neurophysiol. 2007;118(12):2765–73. 17911042 10.1016/j.clinph.2007.07.028

[41] Cohen MX. Analyzing neural time series data: theory and practice. Cambridge: MIT Press; 2014.

[42] Delorme A, Makeig S. EEGLAB: an open source toolbox for analysis of single-trial EEG dynamics including independent component analysis. J Neurosci Methods. 2004;134(1):9–21. 15102499 10.1016/j.jneumeth.2003.10.009

[43] Luck SJ. An introduction to the event-related potential technique. 2nd ed. Cambridge: MIT Press; 2014.

[44] Urigüen JA, Garcia-Zapirain B. EEG artifact removal-state-of-the-art and guidelines. J Neural Eng. 2015;12(3):031001. 25834104 10.1088/1741-2560/12/3/031001

[45] Kemarat S, Theanthong A, Vongjaturapat N. Development and validation of a Thai version of the Athletics Coping Skills Inventory. J Exerc Sport Sci. 2015;11(7):8.

[46] Gelman A, Hill J. Data analysis using regression and multilevel/hierarchical models. Cambridge: Cambridge University Press; 2007.

[47] Schielzeth H, Dingemanse NJ, Nakagawa S, Westneat DF, Allegue H, Teplitsky C, et al. Robustness of linear mixed-effects models to violations of distributional assumptions. Methods Ecol Evol. 2020;11(9):1141–52.

[48] Blanca Mena MJ, Alarcón Postigo R, Arnau Gras J, Bono Cabré R, Bendayan R. Non-normal data: Is ANOVA still a valid option? Psicothema. 2017;29(4):l552–557. 10.7334/psicothema2016.383 29048317

[49] Schielzeth H, Nakagawa S. Nested by design: model fitting and interpretation in a mixed model era. Methods Ecol Evol. 2013;4(1):14–24.

[50] Cohen J. Statistical power analysis for the behavioral sciences. 2nd ed. New York: Routledge; 2013.

[51] Mukaka MM. A guide to appropriate use of correlation coefficient in medical research. Malawi Med J. 2012;24(3):69–71. 23638278 PMC3576830

[52] Nunez PL, Srinivasan R. Electric fields of the brain: the neurophysics of EEG. 2nd ed. Oxford: Oxford University Press; 2006.

[53] Del Percio C, Iacoboni M, Lizio R, Marzano N, Infarinato F, Vecchio F, et al. Functional coupling of parietal alpha rhythms is enhanced in athletes before visuomotor performance: a coherence electroencephalographic study. Neuroscience. 2011;175:198–211. 21144884 10.1016/j.neuroscience.2010.11.031

[54] Li L, Smith DM. Neural efficiency in athletes: a systematic review. Front Behav Neurosci. 2021;15:698555. 34421553 10.3389/fnbeh.2021.698555 PMC8374331

[55] Makeig S, Debener S, Onton J, Delorme A. Mining event-related brain dynamics. Trends Cogn Sci. 2004;8(5):204–10. 15120678 10.1016/j.tics.2004.03.008

[56] Saggar M, King BG, Zanesco AP, MacLean KA, Aichele SR, Jacobs TL, et al. Intensive training induces longitudinal changes in meditation state-related EEG oscillatory activity. Front Hum Neurosci. 2012;6:256. 22973218 10.3389/fnhum.2012.00256 PMC3437523

[57] Van Ede F, De Lange F, Jensen O, Maris E. Orienting attention to an upcoming tactile event involves a spatially and temporally specific modulation of sensorimotor alpha-and beta-band oscillations. J Neurosci. 2011;31(6):2016–24. 21307240 10.1523/JNEUROSCI.5630-10.2011 PMC6633042

[58] Kopell N, Kramer MA, Malerba P, Whittington MA. Are different rhythms good for different functions? Front Hum Neurosci. 2010;4:187. 21103019 10.3389/fnhum.2010.00187 PMC2987659

[59] Miller EK, Lundqvist M, Bastos AM. Working Memory 2 0 Neuron. 2018;100(2):463–75. 10.1016/j.neuron.2018.09.023 PMC8112390 30359609

[60] Palacios-García I, Silva J, Villena-González M, Campos-Arteaga G, Artigas-Vergara C, Luarte N, et al. Increase in beta power reflects attentional top-down modulation after psychosocial stress induction. Front Hum Neurosci. 2021;15:630813. 33833671 10.3389/fnhum.2021.630813 PMC8021732

[61] Patnaik S, Pandey P, Arun I, Yadav G, Miyapuram KP, Lomas D, editors. EEG spectral correlates of rapid and deep slow breathing states and classification using ML. 2022 IEEE Region 10 Symposium (TENSYMP); 2022: IEEE.

[62] Park J, Moghaddam B. Impact of anxiety on prefrontal cortex encoding of cognitive flexibility. Neuroscience. 2017;345:193–202. 27316551 10.1016/j.neuroscience.2016.06.013 PMC5159328

[63] Blair RJ. The roles of orbital frontal cortex in the modulation of antisocial behavior. Biosocial Theor crime. 2017:423–33. 10.1016/S0278-2626(03)00276-8 15134853

[64] Cavanagh JF, Shackman AJ. Frontal midline theta reflects anxiety and cognitive control: meta-analytic evidence. J physiology-Paris. 2015;109(1–3):3–15. 10.1016/j.jphysparis.2014.04.003 PMC4213310 24787485

[65] Stujenske JM, Likhtik E, Topiwala MA, Gordon JA. Fear and safety engage competing patterns of theta-gamma coupling in the basolateral amygdala. Neuron. 2014;83(4):919–33. 25144877 10.1016/j.neuron.2014.07.026 PMC4141236

[66] Cavanagh JF, Frank MJ. Frontal theta as a mechanism for cognitive control. Trends Cogn Sci. 2014;18(8):414–21. 24835663 10.1016/j.tics.2014.04.012 PMC4112145

[67] Karalis N, Sirota A. Breathing coordinates cortico-hippocampal dynamics in mice during offline states. Nat Commun. 2022;13(1):467. 35075139 10.1038/s41467-022-28090-5 PMC8786964

[68] Tort AB, Brankačk J, Draguhn A. Respiration-entrained brain rhythms are global but often overlooked. Trends Neurosci. 2018;41(4):186–97. 29429805 10.1016/j.tins.2018.01.007

[69] Szulczewski MT. Training of paced breathing at 0.1 Hz improves CO2 homeostasis and relaxation during a paced breathing task. PLoS ONE. 2019;14(6):e0218550. 31220170 10.1371/journal.pone.0218550 PMC6586331

[70] Migliaccio GM, Russo L, Maric M, Padulo J. Sports performance and breathing rate: What is the connection? A narrative review on breathing strategies. Sports. 2023;11(5):103. 37234059 10.3390/sports11050103 PMC10224217

[71] Cheng KS, Han RPS, Lee PF. Neurophysiological study on the effect of various short durations of deep breathing: A randomized controlled trial. Respir Physiol Neurobiol. 2018;249:23–31. 29294356 10.1016/j.resp.2017.12.008

[72] Aftanas LI, Golocheikine SA. Human anterior and frontal midline theta and lower alpha reflect emotionally positive state and internalized attention: high-resolution EEG investigation of meditation. Neurosci Lett. 2001;310(1):57–60. 11524157 10.1016/s0304-3940(01)02094-8

[73] Goldin PR, Ziv M, Jazaieri H, Hahn K, Heimberg R, Gross JJ. Impact of cognitive behavioral therapy for social anxiety disorder on the neural dynamics of cognitive reappraisal of negative self-beliefs: randomized clinical trial. JAMA Psychiatry. 2013;70(10):1048–56. 23945981 10.1001/jamapsychiatry.2013.234 PMC4141477

[74] Bellomo E, Cooke A, Gallicchio G, Ring C, Hardy J. Mind and body: Psychophysiological profiles of instructional and motivational self-talk. Psychophysiology. 2020;57(9):e13586. 32412145 10.1111/psyp.13586

[75] Chu TL, Myers B. A novel investigation of NCAA athletes’ psychological skill profiles through a person-centered approach. High Ability Stud. 2025;36(1):1–15.

[76] Van Raalte JL, Vincent A. Self-talk in sport and performance. Oxford: Oxford University Press; 2017.

[77] Allegretta RA, Rovelli K, Balconi M, editors. The role of emotion regulation and awareness in psychosocial stress: an EEG-psychometric correlational study. Healthcare: MDPI; 2024. 10.3390/healthcare12151491 PMC11312088 39120194

[78] Lapomarda G, Valer S, Job R, Grecucci A. Built to last: Theta and delta changes in resting-state EEG activity after regulating emotions. Brain Behav. 2022;12(6):e2597. 35560984 10.1002/brb3.2597 PMC9226824

[79] Pscherer C, Mückschel M, Summerer L, Bluschke A, Beste C. On the relevance of EEG resting theta activity for the neurophysiological dynamics underlying motor inhibitory control. Hum Brain Mapp. 2019;40(14):4253–65. 31219652 10.1002/hbm.24699 PMC6865515

[80] Abid A, Hamrick HC, Mach RJ, Hager NM, Judah MR. Emotion regulation strategies explain associations of theta and Beta with positive affect. Psychophysiology. 2025;62(1):e14745. 39690435 10.1111/psyp.14745 PMC11652703

[81] Lum JAG, Byrne LK, Barhoun P, Hyde C, Hill AT, Enticott PG, et al. Resting state electroencephalography power correlates with individual differences in implicit sequence learning. Eur J Neurosci. 2023;58(3):2838–52. 37317510 10.1111/ejn.16059

[82] LaFave SE, Granbom M, Cudjoe TKM, Gottsch A, Shorb G, Szanton SL. Attention control group activities and perceived benefit in a trial of a behavioral intervention for older adults. Res Nurs Health. 2019;42(6):476–82. 31647125 10.1002/nur.21992 PMC6858509

