# Supplementary material for: Effects of a 4-week combined breathing control and self-talk intervention on neural activity and coping skills in athletes: a randomized control trial
Source: BMC Psychol. 2026 Jun 5;14:1159. doi: 10.1186/s40359-026-04814-w (PMC13455456; doi:10.1186/s40359-026-04814-w)
Supplement: Supplementary file 1 [file 40359_2026_4814_MOESM1_ESM.docx]

Supplementary Files

**Supplementary A:** Summary of Mann-Whitney U Test: Analysis of Inter-Group Differences

|  | Group | N | Mean | SD | p-value |
| --- | --- | --- | --- | --- | --- |
| Age  (year) | Experimental | 13 | 22.08 | 3.62 | 0.141 |
|  | Control | 13 | 19.69 | 2.25 |  |
| Weight  (kg) | Experimental | 13 | 60.62 | 12.44 | 0.136 |
|  | Control | 13 | 55.31 | 9.67 |  |
| Height  (cm) | Experimental | 13 | 167.46 | 9.76 | 0.456 |
|  | Control | 13 | 165.23 | 8.98 |  |
| Experience  (year) | Experimental | 13 | 4.15 | 2.96 | 0.640 |
|  | Control | 13 | 3.23 | 2.05 |  |

**Supplementary B:** Primary Sporting Disciplines of Participants.

| Primary sport / Sex | Group | | Total |
| --- | --- | --- | --- |
|  | **Experimental** | **Control** |  |
| Swimming / Male | 2 | 2 | 4 |
| Swimming / Female | 4 | 3 | 7 |
| Gymnast / Male | 1 | 2 | 3 |
| Gymnast / Female | 6 | 6 | 12 |
| Total | 13 | 13 | 26 |

**Supplementary File C:** Descriptive statistics and Shapiro-Wilk normality test for EEG variables across groups and time points.

|  | **Brain areas** | **Times** | **Groups** | **Mean** | **95%CI mean** | | **SD** | **Median** | **Shapiro-Wilk p** |
| --- | --- | --- | --- | --- | --- | --- | --- | --- | --- |
|  |  |  |  |  | **Lower bound** | **Upper bound** |  |  |  |
| Delta Power | Pre-frontal | Pre | Exp | 143 | 96.6 | 190 | 77.1 | 112 | .080 |
|  |  |  | Con | 133 | 104 | 162 | 47.6 | 128 | .228 |
|  |  | Post | Exp | 140 | 78.3 | 202 | 102 | 106 | .024* |
|  |  |  | Con | 158 | 122 | 194 | 59.7 | 132 | .129 |
|  | Frontal | Pre | Exp | 72.8 | 53.0 | 92.6 | 32.8 | 76.5 | .223 |
|  |  |  | Con | 61.8 | 50.4 | 73.2 | 18.9 | 59.6 | .871 |
|  |  | Post | Exp | 64.6 | 43.4 | 85.7 | 35.0 | 48.2 | .065 |
|  |  |  | Con | 79.4 | 59.9 | 99.0 | 32.3 | 75.3 | .357 |
|  | Temporal | Pre | Exp | 57.9 | 34.2 | 81.7 | 39.3 | 51.4 | .010* |
|  |  |  | Con | 39.0 | 28.2 | 49.8 | 17.8 | 36.9 | .705 |
|  |  | Post | Exp | 40.8 | 23.4 | 58.2 | 28.8 | 29.5 | .079 |
|  |  |  | Con | 52.2 | 35.1 | 69.3 | 28.3 | 41.9 | .013* |
|  | Central | Pre | Exp | 52.1 | 40.0 | 64.2 | 20.0 | 53.0 | .613 |
|  |  |  | Con | 42.5 | 31.2 | 53.9 | 18.8 | 35.1 | .005* |
|  |  | Post | Exp | 45.7 | 28.6 | 62.8 | 28.3 | 33.9 | .117 |
|  |  |  | Con | 51.8 | 38.2 | 65.4 | 22.5 | 42.5 | .169 |
|  | Parietal | Pre | Exp | 47.9 | 35.9 | 60.0 | 19.9 | 43.0 | .137 |
|  |  |  | Con | 39.7 | 27.9 | 51.6 | 19.6 | 34.3 | .015* |
|  |  | Post | Exp | 40.7 | 24.4 | 57.0 | 27.0 | 28.8 | .106 |
|  |  |  | Con | 44.8 | 34.3 | 55.3 | 17.4 | 41.5 | .471 |
|  | Occipital | Pre | Exp | 48.7 | 34.3 | 63.2 | 23.9 | 44.5 | .640 |
|  |  |  | Con | 33.3 | 23.6 | 43.0 | 16.1 | 30.6 | .003* |
|  |  | Post | Exp | 42.6 | 24.0 | 61.1 | 30.7 | 35.1 | .035* |
|  |  |  | Con | 40.8 | 34.6 | 46.9 | 10.2 | 44.2 | .466 |
| Theta Power | Pre-frontal | Pre | Exp | 4.92 | 3.68 | 6.17 | 2.06 | 4.96 | .951 |
|  |  |  | Con | 5.82 | 4.92 | 6.73 | 1.49 | 5.57 | .094 |
|  |  | Post | Exp | 10.6 | 9.54 | 11.8 | 1.84 | 10.1 | .150 |
|  |  |  | Con | 5.61 | 4.16 | 7.06 | 2.40 | 5.11 | .634 |
|  | Frontal | Pre | Exp | 5.72 | 4.66 | 6.78 | 1.75 | 5.57 | .881 |
|  |  |  | Con | 6.40 | 5.62 | 7.19 | 1.30 | 6.52 | .972 |
|  |  | Post | Exp | 7.08 | 6.12 | 8.03 | 1.57 | 6.87 | .830 |
|  |  |  | Con | 5.24 | 3.95 | 6.54 | 2.14 | 4.76 | .162 |
|  | Temporal | Pre | Exp | 3.42 | 2.89 | 3.95 | .880 | 3.57 | .469 |
|  |  |  | Con | 3.44 | 2.77 | 4.11 | 1.10 | 3.07 | .140 |
|  |  | Post | Exp | 3.57 | 2.28 | 4.86 | 2.13 | 2.62 | .036* |
|  |  |  | Con | 3.84 | 3.10 | 4.58 | 1.22 | 3.48 | .180 |
|  | Central | Pre | Exp | 6.53 | 5.62 | 7.44 | 1.50 | 7.15 | .134 |
|  |  |  | Con | 5.79 | 4.93 | 6.65 | 1.42 | 5.68 | .921 |
|  |  | Post | Exp | 6.01 | 4.14 | 7.89 | 3.10 | 4.27 | .037* |
|  |  |  | Con | 6.23 | 5.20 | 7.25 | 1.70 | 5.64 | .124 |
|  | Parietal | Pre | Exp | 5.62 | 4.75 | 6.48 | 1.44 | 5.56 | .525 |
|  |  |  | Con | 4.54 | 3.85 | 5.24 | 1.15 | 4.50 | .616 |
|  |  | Post | Exp | 5.07 | 3.37 | 6.78 | 2.82 | 3.61 | .021 |
|  |  |  | Con | 5.06 | 4.16 | 5.95 | 1.49 | 4.49 | .567 |
|  | Occipital | Pre | Exp | 5.27 | 4.32 | 6.23 | 1.58 | 6.06 | .034* |
|  |  |  | Con | 3.66 | 2.84 | 4.47 | 1.35 | 3.11 | .025* |
|  |  | Post | Exp | 5.22 | 3.28 | 7.15 | 3.20 | 3.14 | .011* |
|  |  |  | Con | 4.30 | 3.65 | 4.95 | 1.07 | 3.92 | .278 |
| Alpha Power | Pre-frontal | Pre | Exp | 8.91 | 4.06 | 13.8 | 8.92 | 5.23 | <.001* |
|  |  |  | Con | 11.3 | 5.86 | 16.7 | 9.97 | 6.43 | .003* |
|  |  | Post | Exp | 8.15 | 4.58 | 11.7 | 6.58 | 4.69 | .001* |
|  |  |  | Con | 11.2 | 5.38 | 17.1 | 10.7 | 6.91 | .001* |
|  | Frontal | Pre | Exp | 8.24 | 3.88 | 12.6 | 8.02 | 4.24 | <.001* |
|  |  |  | Con | 10.8 | 5.58 | 15.9 | 9.52 | 7.20 | .004* |
|  |  | Post | Exp | 8.78 | 4.69 | 12.9 | 7.52 | 4.82 | .002* |
|  |  |  | Con | 10.3 | 5.18 | 15.3 | 9.35 | 6.95 | .001* |
|  | Temporal | Pre | Exp | 4.62 | 2.69 | 6.55 | 3.55 | 4.02 | .046* |
|  |  |  | Con | 5.79 | 3.55 | 8.04 | 4.13 | 4.85 | .031* |
|  |  | Post | Exp | 4.75 | 2.91 | 6.59 | 3.39 | 3.10 | .008* |
|  |  |  | Con | 5.43 | 3.47 | 7.39 | 3.61 | 4.06 | .023* |
|  | Central | Pre | Exp | 10.9 | 4.67 | 17.1 | 11.4 | 4.88 | .002* |
|  |  |  | Con | 11.8 | 7.01 | 16.6 | 8.84 | 8.48 | .085 |
|  |  | Post | Exp | 11.6 | 6.47 | 16.7 | 9.38 | 7.32 | .015* |
|  |  |  | Con | 11.5 | 6.59 | 16.5 | 9.11 | 8.07 | .053 |
|  | Parietal | Pre | Exp | 12.4 | 3.35 | 21.4 | 16.6 | 4.45 | <.001* |
|  |  |  | Con | 10.8 | 6.10 | 15.4 | 8.58 | 7.12 | .036* |
|  |  | Post | Exp | 12.7 | 6.73 | 18.7 | 11.0 | 8.58 | .014* |
|  |  |  | Con | 11.8 | 5.69 | 18.0 | 11.3 | 7.30 | .005* |
|  | Occipital | Pre | Exp | 12.3 | 1.85 | 22.8 | 19.3 | 4.25 | <.001* |
|  |  |  | Con | 10.1 | 4.79 | 15.5 | 9.86 | 5.10 | .003* |
|  |  | Post | Exp | 10.8 | 4.77 | 16.7 | 11.0 | 7.25 | .001* |
|  |  |  | Con | 9.66 | 4.48 | 14.8 | 9.53 | 6.39 | .004* |
| Beta Power | Pre-frontal | Pre | Exp | 10.6 | 9.18 | 12.1 | 2.42 | 10.6 | .389 |
|  |  |  | Con | 7.62 | 5.42 | 9.81 | 3.63 | 6.30 | .060 |
|  |  | Post | Exp | 5.59 | 4.53 | 6.66 | 1.76 | 5.10 | .232 |
|  |  |  | Con | 7.84 | 6.61 | 9.07 | 2.04 | 7.48 | .042* |
|  | Frontal | Pre | Exp | 12.8 | 11.7 | 14.0 | 1.94 | 12.5 | .583 |
|  |  |  | Con | 11.5 | 10.2 | 12.8 | 2.18 | 12.0 | .225 |
|  |  | Post | Exp | 6.97 | 5.97 | 7.97 | 1.66 | 7.42 | .211 |
|  |  |  | Con | 12.0 | 11.3 | 12.8 | 1.21 | 12.3 | .413 |
|  | Temporal | Pre | Exp | 9.10 | 5.70 | 12.5 | 5.63 | 7.44 | .238 |
|  |  |  | Con | 7.67 | 4.90 | 10.4 | 4.59 | 6.24 | .070 |
|  |  | Post | Exp | 6.28 | 4.37 | 8.20 | 3.17 | 5.60 | .018* |
|  |  |  | Con | 7.80 | 5.23 | 10.4 | 4.25 | 5.66 | .024* |
|  | Central | Pre | Exp | 6.31 | 4.99 | 7.63 | 2.18 | 5.72 | .571 |
|  |  |  | Con | 5.91 | 4.60 | 7.23 | 2.17 | 5.35 | .684 |
|  |  | Post | Exp | 6.06 | 4.59 | 7.53 | 2.44 | 5.98 | .995 |
|  |  |  | Con | 6.77 | 5.53 | 8.01 | 2.05 | 6.24 | .295 |
|  | Parietal | Pre | Exp | 6.70 | 5.19 | 8.20 | 2.49 | 5.95 | .343 |
|  |  |  | Con | 5.97 | 4.67 | 7.26 | 2.14 | 5.87 | .676 |
|  |  | Post | Exp | 5.78 | 4.39 | 7.17 | 2.30 | 6.40 | .658 |
|  |  |  | Con | 6.61 | 5.20 | 8.01 | 2.32 | 6.48 | .557 |
|  | Occipital | Pre | Exp | 6.98 | 4.71 | 9.25 | 3.76 | 6.26 | 324 |
|  |  |  | Con | 6.11 | 4.65 | 7.57 | 2.42 | 5.87 | .227 |
|  |  | Post | Exp | 6.30 | 4.31 | 8.29 | 3.30 | 5.92 | .593 |
|  |  |  | Con | 6.01 | 4.59 | 7.43 | 2.36 | 5.19 | .040* |

* Significance level seat at p < 0.05

Abbreviations: Exp = Experimental group; Con = Control group; Pre = Recording data at 3 months (pre-intervention);
 Post = Recording data at 1 week (post-intervention); SD = Standard deviatio

**Supplementary File D:** Descriptive statistics and Shapiro-Wilk normality test for ACSI-28 subscales across groups and time points.

| **Subscales** | **Times** | **Groups** | **Mean** | **95%CI mean** | | **SD** | **Median** | **Shapiro-Wilk**  **p** |
| --- | --- | --- | --- | --- | --- | --- | --- | --- |
|  |  |  |  | **Lower bound** | **Upper bound** |  |  |  |
| Coping with adversity | Pre | Exp | 7.50 | 6.07 | 9.32 | 2.69 | 8 | .202 |
|  |  | Con | 6.15 | 5.20 | 7.10 | 1.57 | 5 | .002* |
|  | Post | Exp | 10.2 | 9.48 | 11.0 | 1.24 | 10 | .353 |
|  |  | Con | 6.54 | 5.31 | 7.76 | 2.03 | 7 | .373 |
| Coachability | Pre | Exp | 7.85 | 6.62 | 9.08 | 2.03 | 8 | .106 |
|  |  | Con | 5.69 | 4.63 | 6.75 | 1.75 | 6 | .468 |
|  | Post | Exp | 8.15 | 7.30 | 9.00 | 1.41 | 8 | .049* |
|  |  | Con | 5.92 | 4.98 | 6.86 | 1.55 | 6 | .332 |
| Concentration | Pre | Exp | 7.23 | 5.71 | 8.75 | 2.52 | 8 | .426 |
|  |  | Con | 6.46 | 8.78 | 7.30 | 1.39 | 7 | .215 |
|  | Post | Exp | 9.77 | 5.62 | 10.8 | 1.64 | 10 | .084 |
|  |  | Con | 6.77 | 5.81 | 7.73 | 1.59 | 7 | .716 |
| Confidence and achievement motivation | Pre | Exp | 7.85 | 6.86 | 8.83 | 1.63 | 8 | .653 |
|  |  | Con | 7.00 | 5.95 | 8.05 | 1.73 | 8 | <.001* |
|  | Post | Exp | 10.2 | 9.38 | 10.9 | 1.28 | 10 | .070 |
|  |  | Con | 7.23 | 5.73 | 8.73 | 2.49 | 8 | .027* |
| Goal setting / Mental preparation | Pre | Exp | 8.23 | 6.95 | 9.52 | 2.13 | 8 | .152 |
|  |  | Con | 7.69 | 6.72 | 8.66 | 1.60 | 8 | .189 |
|  | Post | Exp | 10.6 | 9.78 | 11.5 | 1.39 | 11 | .010* |
|  |  | Con | 7.54 | 6.09 | 8.99 | 2.40 | 8 | .526 |
| Peaking under pressure | Pre | Exp | 5.69 | 3.94 | 7.44 | 2.90 | 6 | .335 |
|  |  | Con | 4.15 | 3.00 | 5.31 | 1.91 | 4 | .742 |
|  | Post | Exp | 8.15 | 7.08 | 9.22 | 1.77 | 9 | .035* |
|  |  | Con | 4.62 | 3.55 | 5.68 | 1.76 | 4 | .023* |
| Freedom from worry | Pre | Exp | 3.23 | 2.41 | 4.05 | 1.36 | 3 | .643 |
|  |  | Con | 3.62 | 2.89 | 4.34 | 1.19 | 4 | .105 |
|  | Post | Exp | 5.69 | 4.16 | 7.22 | 2.53 | 6 | .253 |
|  |  | Con | 4.85 | 3.64 | 6.05 | 1.99 | 5 | .032* |

* Significance level seat at p < 0.05

Abbreviations: Exp = Experimental group; Con = Control group; Pre = Recording data at 3 months (pre-intervention);
 Post = Recording data at 1 week (post-intervention); SD = Standard deviation

**Supplementary File E:** Residual summary statistics, Q-Q plot, and histogram

|  | **GLMM residual of EEG** | | | |
| --- | --- | --- | --- | --- |
|  | **Delta Power** | **Theta Power** | **Alpha Power** | **Beta Power** |
| Mean | -.075 | -.040 | -.211 | -.049 |
| Median | -.111 | -.049 | -.397 | -.053 |
| Standard Deviation | .473 | .346 | .777 | .386 |
| Minimum | -1.24 | -1.15 | -1.68 | -1.20 |
| Maximum | 1.22 | .896 | 2.53 | .971 |
| Skewness | .322 | -.019 | .814 | -.050 |
| Kurtosis | -.018 | .149 | .315 | .135 |
| Shapiro-Wilk (W) | .989 | .996 | .950 | .995 |
| Shapiro-Wilk (p) | .022 | .563 | <.001 | .410 |

Residual diagnostics (histogram and Q-Q plot) for delta power

Residual diagnostics (histogram and Q-Q plot) for theta power

Residual diagnostics (histogram and Q-Q plot) for alpha power

Residual diagnostics (histogram and Q-Q plot) for beta power

**Supplementary File F:** Correlations between EEG power spectral density and ACSI-28 subscales score in the **experimental group**

| **Experimental group** | | | | | | | | | |
| --- | --- | --- | --- | --- | --- | --- | --- | --- | --- |
| **Power** | **Areas** | **Correlation** | **Coping** | **Coach** | **Concent** | **Confident** | **Goal** | **Peak** | **Free** |
| Delta | Pre-frontal | Spearman’s rho | -.229 | .040 | -.514 | -.621 | **-.702** | -.368 | -.284 |
|  |  | p-value | .452 | .898 | .072 | .023 | **.007*** | .216 | .347 |
|  |  | p (FDR-adjusted) | .933 | .898 | .432 | .138 | **.042*** | .514 | .885 |
|  | Frontal | Spearman’s rho | -.032 | .257 | -.257 | -.376 | -.495 | -.199 | .053 |
|  |  | p-value | .918 | .396 | .397 | .205 | .085 | .514 | .864 |
|  |  | p (FDR-adjusted) | .933 | .635 | .451 | .410 | .165 | .514 | .885 |
|  | Temporal | Spearman’s rho | -.293 | -.192 | -.425 | -.396 | -.405 | -.398 | -.045 |
|  |  | p-value | .332 | .529 | .147 | .181 | .170 | .178 | .885 |
|  |  | p (FDR-adjusted) | .933 | .635 | .441 | .410 | .170 | .514 | .885 |
|  | Central | Spearman’s rho | -.026 | .300 | -.229 | .242 | -.464 | -.199 | .064 |
|  |  | p-value | .933 | .320 | .451 | .425 | .110 | .514 | .835 |
|  |  | p (FDR-adjusted) | .933 | .635 | .451 | .510 | .165 | .514 | .885 |
|  | Parietal | Spearman’s rho | -.058 | .237 | -.290 | -.245 | -.552 | -.282 | -.078 |
|  |  | p-value | .851 | .435 | .336 | .420 | .051 | .350 | .800 |
|  |  | p (FDR-adjusted) | .933 | .635 | .451 | .510 | .153 | .514 | .885 |
|  | Occipital | Spearman’s rho | -.049 | .209 | -.232 | -.139 | -.430 | -.307 | -.064 |
|  |  | p-value | .873 | .493 | .446 | .650 | .142 | .307 | .835 |
|  |  | p (FDR-adjusted) | .933 | .635 | .451 | .650 | .170 | .514 | .885 |
| Theta | Pre-frontal | Spearman’s rho | .252 | .345 | .304 | .513 | .526 | -.174 | .184 |
|  |  | p-value | .406 | .249 | .313 | .073 | .065 | .569 | .548 |
|  |  | p (FDR-adjusted) | .780 | .498 | .576 | .438 | .390 | .569 | .702 |
|  | Frontal | Spearman’s rho | .458 | .497 | .227 | .410 | .241 | -.194 | .237 |
|  |  | p-value | .116 | .084 | .457 | .165 | .429 | .526 | .436 |
|  |  | p (FDR-adjusted) | .696 | .252 | .576 | .495 | .515 | .569 | .702 |
|  | Temporal | Spearman’s rho | .180 | .497 | .019 | .234 | -.040 | -.183 | .114 |
|  |  | p-value | .557 | .084 | .050 | .442 | .898 | .550 | .710 |
|  |  | p (FDR-adjusted) | .780 | .252 | .950 | .884 | .898 | .569 | .710 |
|  | Central | Spearman’s rho | -.139 | .090 | -.282 | .017 | -.334 | -.249 | -.176 |
|  |  | p-value | .650 | .769 | .351 | .957 | .265 | .412 | .566 |
|  |  | p (FDR-adjusted) | .780 | .923 | .576 | .964 | .437 | .569 | .702 |
|  | Parietal | Spearman’s rho | -.075 | .192 | -.215 | .014 | -.317 | -.238 | -.167 |
|  |  | p-value | .807 | .529 | .480 | .964 | .291 | .434 | .585 |
|  |  | p (FDR-adjusted) | .807 | .794 | .576 | .964 | .437 | .569 | .702 |
|  | Occipital | Spearman’s rho | -.206 | .006 | -.365 | -.100 | -.348 | -.365 | -.206 |
|  |  | p-value | .500 | .985 | .221 | .744 | .244 | .220 | .499 |
|  |  | p (FDR-adjusted) | .780 | .985 | .576 | .964 | .437 | .569 | .702 |
| Alpha | Pre-frontal | Spearman’s rho | .009 | .328 | .169 | -.181 | .158 | .019 | .457 |
|  |  | p-value | .978 | .274 | .582 | .554 | .605 | .950 | .117 |
|  |  | p (FDR-adjusted) | .978 | .563 | .979 | .665 | .941 | .950 | .416 |
|  | Frontal | Spearman’s rho | -.087 | .300 | .055 | -.279 | .031 | -.061 | .332 |
|  |  | p-value | .778 | .320 | .858 | .357 | .920 | .843 | .269 |
|  |  | p (FDR-adjusted) | .978 | .563 | .979 | .665 | .941 | .950 | .416 |
|  | Temporal | Spearman’s rho | .064 | .017 | .171 | .042 | .325 | -.160 | .109 |
|  |  | p-value | .836 | .956 | .576 | .892 | .278 | .601 | .724 |
|  |  | p (FDR-adjusted) | .978 | .956 | .979 | .892 | .941 | .950 | .724 |
|  | Central | Spearman’s rho | -.206 | .268 | .008 | -.362 | -.023 | -.111 | .248 |
|  |  | p-value | .500 | .375 | .979 | .224 | .941 | .719 | .414 |
|  |  | p (FDR-adjusted) | .978 | .563 | .979 | .665 | .941 | .950 | .497 |
|  | Parietal | Spearman’s rho | -.136 | .277 | .050 | -.379 | -.037 | -.044 | .326 |
|  |  | p-value | .657 | .360 | .872 | .202 | .905 | .886 | .277 |
|  |  | p (FDR-adjusted) | .978 | .563 | .979 | .665 | .941 | .950 | .416 |
|  | Occipital | Spearman’s rho | .014 | .068 | .108 | -.228 | .147 | .047 | .334 |
|  |  | p-value | .963 | .826 | .726 | .453 | .631 | .879 | .264 |
|  |  | p (FDR-adjusted) | .978 | .956 | .979 | .665 | .941 | .950 | .416 |
| Beta | Pre-frontal | Spearman’s rho | -.096 | -.122 | -.296 | -.217 | -.286 | -.321 | -.560 |
|  |  | p-value | .756 | .693 | .327 | .476 | .344 | .285 | .047 |
|  |  | p (FDR-adjusted) | .948 | .912 | .747 | .914 | .413 | .979 | .282 |
|  | Frontal | Spearman’s rho | -.325 | -.102 | -.124 | -.223 | -.076 | -.083 | -.106 |
|  |  | p-value | .279 | .741 | .686 | .464 | .804 | .788 | .731 |
|  |  | p (FDR-adjusted) | .948 | .912 | .747 | .914 | .804 | .979 | .877 |
|  | Temporal | Spearman’s rho | .151 | .034 | -.099 | -.195 | -.348 | .008 | -.354 |
|  |  | p-value | .623 | .912 | .747 | .523 | .244 | .979 | .236 |
|  |  | p (FDR-adjusted) | .948 | .912 | .747 | .914 | .413 | .979 | .472 |
|  | Central | Spearman’s rho | .032 | .328 | -.169 | -.042 | -.456 | -.144 | -.011 |
|  |  | p-value | .918 | .274 | .582 | .892 | .118 | .639 | .971 |
|  |  | p (FDR-adjusted) | .948 | .912 | .747 | .914 | .413 | .979 | .971 |
|  | Parietal | Spearman’s rho | .020 | .167 | -.213 | .081 | -.320 | -.033 | -.109 |
|  |  | p-value | .948 | .586 | .485 | .793 | .287 | .914 | .724 |
|  |  | p (FDR-adjusted) | .948 | .912 | .747 | .914 | .413 | .979 | .877 |
|  | Occipital | Spearman’s rho | -.203 | .093 | -.273 | -.033 | -.382 | -.194 | -.387 |
|  |  | p-value | .506 | .762 | .366 | .914 | .198 | .526 | .191 |
|  |  | p (FDR-adjusted) | .948 | .912 | .747 | .914 | .413 | .979 | .472 |

* Significance level seat at p < 0.05

Abbreviation: Coping = Coping with adversity, Coach = Coachability, Concent = Concentration, Confident = Confidence and achievement motivation, Goal = Goal setting/Mental preparation, Peak = Peaking under pressure, Free = Freedom from worry

**Supplementary File G:** Correlations between EEG power spectral density and ACSI-28 subscales score in the **control group**

| **Control group** | | | | | | | | | |
| --- | --- | --- | --- | --- | --- | --- | --- | --- | --- |
| **Power** | **Areas** | **Correlation** | **Coping** | **Coach** | **Concent** | **Confident** | **Goal** | **Peak** | **Free** |
| Delta | Pre-frontal | Spearman’s rho | **-.851** | -.159 | .015 | -.180 | -.109 | -.245 | .257 |
|  |  | p-value | **<.001*** | .604 | .962 | .556 | .722 | .420 | .396 |
|  |  | p (FDR-adjusted) | **.006*** | .950 | .962 | .964 | .993 | .420 | .928 |
|  | Frontal | Spearman’s rho | **-.669** | -.050 | .462 | -.089 | .006 | -.288 | .298 |
|  |  | p-value | **.012*** | .871 | .112 | .773 | .985 | .341 | .323 |
|  |  | p (FDR-adjusted) | **.024*** | .950 | .188 | .964 | .993 | .420 | .928 |
|  | Temporal | Spearman’s rho | -.537 | -.020 | .538 | .053 | .003 | -.305 | -.009 |
|  |  | p-value | .059 | .950 | .058 | .864 | .993 | .312 | .976 |
|  |  | p (FDR-adjusted) | .071 | .950 | .188 | .964 | .993 | .420 | .976 |
|  | Central | Spearman’s rho | **-.700** | -.139 | .448 | -.194 | .185 | -.558 | .223 |
|  |  | p-value | **.008*** | .649 | .125 | .525 | .545 | .048 | .464 |
|  |  | p (FDR-adjusted) | **.024*** | .950 | .188 | .964 | .993 | .219 | .928 |
|  | Parietal | Spearman’s rho | -.559 | -.089 | .489 | -.014 | .264 | -.512 | .022 |
|  |  | p-value | .047 | .772 | .090 | .964 | .384 | .073 | .943 |
|  |  | p (FDR-adjusted) | .071 | .950 | .188 | .964 | .993 | .219 | .976 |
|  | Occipital | Spearman’s rho | -.475 | -.103 | .401 | .172 | .255 | -.273 | .069 |
|  |  | p-value | .101 | .737 | .175 | .574 | .400 | .366 | .823 |
|  |  | p (FDR-adjusted) | .101 | .950 | .210 | .964 | .993 | .420 | .976 |
| Theta | Pre-frontal | Spearman’s rho | .376 | -.003 | -.234 | .022 | -.272 | .544 | .304 |
|  |  | p-value | .205 | .993 | .442 | .943 | .368 | .055 | .312 |
|  |  | p (FDR-adjusted) | .432 | .993 | .530 | .943 | .526 | .330 | .951 |
|  | Frontal | Spearman’s rho | .230 | -.262 | .067 | **.718** | .483 | .028 | -.085 |
|  |  | p-value | .449 | .387 | .827 | **.006*** | .095 | .926 | .783 |
|  |  | p (FDR-adjusted) | .449 | .993 | .827 | **.036*** | .526 | .926 | .951 |
|  | Temporal | Spearman’s rho | -.273 | -.014 | .582 | .424 | .236 | -.196 | -.069 |
|  |  | p-value | .368 | .964 | .037 | .148 | .438 | .520 | .823 |
|  |  | p (FDR-adjusted) | .442 | .993 | .222 | .387 | .526 | .731 | .951 |
|  | Central | Spearman’s rho | -.273 | -.033 | .407 | .338 | .255 | -.225 | -.050 |
|  |  | p-value | .368 | .914 | .168 | .258 | .400 | .460 | .871 |
|  |  | p (FDR-adjusted) | .442 | .993 | .252 | .387 | .526 | .731 | .951 |
|  | Parietal | Spearman’s rho | -.410 | .086 | .439 | .344 | .253 | -.157 | -.019 |
|  |  | p-value | .164 | .779 | .134 | .250 | .405 | .609 | .951 |
|  |  | p (FDR-adjusted) | .432 | .993 | .252 | .387 | .526 | .731 | .951 |
|  | Occipital | Spearman’s rho | -.368 | -.008 | .456 | .111 | .160 | -.179 | .257 |
|  |  | p-value | .216 | .978 | .117 | .718 | .602 | .558 | .396 |
|  |  | p (FDR-adjusted) | .432 | .993 | .252 | .862 | .602 | .731 | .951 |
| Alpha | Pre-frontal | Spearman’s rho | -.242 | -.332 | .041 | -.386 | .123 | -.307 | -.069 |
|  |  | p-value | .426 | .268 | .894 | .193 | .688 | .307 | .823 |
|  |  | p (FDR-adjusted) | .631 | .322 | .894 | .852 | .688 | .468 | .976 |
|  | Frontal | Spearman’s rho | -.194 | -.485 | .140 | -.092 | .519 | -.481 | -.166 |
|  |  | p-value | .526 | .093 | .647 | .766 | .069 | .096 | .587 |
|  |  | p (FDR-adjusted) | .631 | .242 | .894 | .871 | .207 | .468 | .976 |
|  | Temporal | Spearman’s rho | -.132 | -.452 | -.097 | -.175 | .216 | -.196 | .009 |
|  |  | p-value | .667 | .121 | .754 | .568 | .478 | .520 | .976 |
|  |  | p (FDR-adjusted) | .667 | .242 | .894 | .852 | .574 | .520 | .976 |
|  | Central | Spearman’s rho | -.250 | -.483 | .246 | -.050 | .519 | -.370 | .013 |
|  |  | p-value | .410 | .095 | .418 | .871 | .069 | .213 | .968 |
|  |  | p (FDR-adjusted) | .631 | .242 | .894 | .871 | .207 | .468 | .976 |
|  | Parietal | Spearman’s rho | -.287 | -.265 | .187 | -.205 | .244 | -.305 | -.035 |
|  |  | p-value | .342 | .382 | .540 | .501 | .422 | .312 | -.911 |
|  |  | p (FDR-adjusted) | .631 | .382 | .894 | .852 | .574 | .468 | .976 |
|  | Occipital | Spearman’s rho | -.225 | -.402 | -.143 | -.261 | .255 | -.228 | .019 |
|  |  | p-value | .460 | .174 | .640 | .390 | .400 | .454 | .951 |
|  |  | p (FDR-adjusted) | .631 | .261 | .894 | .852 | .574 | .520 | .976 |
| Beta | Pre-frontal | Spearman’s rho | -.528 | .050 | -.468 | -.125 | -.219 | -.270 | .185 |
|  |  | p-value | .064 | .871 | .107 | .684 | .473 | .372 | .545 |
|  |  | p (FDR-adjusted) | .252 | .964 | .534 | .821 | .993 | .883 | .984 |
|  | Frontal | Spearman’s rho | -.194 | -.218 | -.398 | -.216 | .006 | -.120 | .295 |
|  |  | p-value | .526 | .475 | .178 | .478 | .985 | .697 | .328 |
|  |  | p (FDR-adjusted) | 1.00 | .964 | .534 | .717 | .993 | .883 | .984 |
|  | Temporal | Spearman’s rho | -.497 | -.064 | -.079 | .033 | .020 | -.097 | .377 |
|  |  | p-value | .084 | .835 | .798 | .914 | .949 | .753 | .205 |
|  |  | p (FDR-adjusted) | .252 | .964 | .835 | .914 | .993 | .883 | .984 |
|  | Central | Spearman’s rho | -.006 | .126 | -.143 | .397 | -.003 | -.122 | -.006 |
|  |  | p-value | .985 | .683 | .640 | .180 | .993 | .690 | .984 |
|  |  | p (FDR-adjusted) | 1.00 | .964 | .835 | .360 | .993 | .883 | .984 |
|  | Parietal | Spearman’s rho | .000 | .014 | -.064 | .546 | .104 | -.046 | -.056 |
|  |  | p-value | 1.00 | .964 | .835 | .053 | .736 | .883 | .855 |
|  |  | p (FDR-adjusted) | 1.00 | .964 | .835 | .318 | .993 | .883 | .984 |
|  | Occipital | Spearman’s rho | .067 | .014 | .082 | .397 | .095 | -.253 | -.028 |
|  |  | p-value | .827 | .964 | .790 | .180 | .757 | .404 | .927 |
|  |  | p (FDR-adjusted) | 1.00 | .964 | .835 | .360 | .993 | .883 | .984 |

* Significance level seat at p < 0.05

Abbreviation: Coping = Coping with adversity, Coach = Coachability, Concent = Concentration, Confident = Confidence and achievement motivation, Goal = Goal setting/Mental preparation, Peak = Peaking under pressure, Free = Freedom from worry
